# Effects of arm-crank exercise on cardiovascular function, functional capacity, cognition and quality of life in patients with peripheral artery disease: Study protocol for a randomized controlled trial

**DOI:** 10.1371/journal.pone.0267849

**Published:** 2022-05-05

**Authors:** Hélcio Kanegusuku, Marília Almeida Correia, Paulo Longano, Raphael Mendes Ritti-Dias, Nelson Wolosker, Gabriel Grizzo Cucato

**Affiliations:** 1 Hospital Israelita Albert Einstein, São Paulo, Brazil; 2 Graduated Program in Medicine, Universidade Nove de Julho, São Paulo, Brazil; 3 Graduated Program in Rehabilitation Sciences, Universidade Nove de Julho, São Paulo, Brazil; 4 Department of Sport, Exercise and Rehabilitation, Northumbria University, Newcastle upon Tyne, United Kingdom; Kurume University School of Medicine, JAPAN

## Abstract

**Background:**

Arm-crank exercise training (ACT) is an alternative exercise strategy for patients with symptomatic peripheral artery disease (PAD) due to the attenuation of pain symptoms during the exercise, as well as the benefits to functional capacity.

**Purpose:**

The aim of this study is to describe the study protocol to analyze the effects of ACT exercise on cardiovascular function, functional capacity, cognition and quality of life in patients with symptomatic PAD.

**Methods:**

This is a three-armed randomized, prospective, single-blind data collection, single-center, controlled study enrolling 45 patients with symptomatic PAD who will be randomized into 3 intervention groups: walking training (WT), ACT and control group. The WT and ACT will perform 2 sessions/week, 15 to 10 sets of 2 to 5 minutes at values of 13 to 15 on the Borg scale. Before and after 12 weeks of intervention, cardiovascular function (ambulatory blood pressure, office blood pressure, central blood pressure, heart rate variability, arterial stiffness and vascular function), functional capacity (six-minute walk test, 2 minute step test, handgrip test, Walking impairment questionnaire, Walking estimated limitation calculated by history, Baltimore activity scale for intermittent claudication, and short physical performance battery), cognition (executive function and memory), and quality of life (vascular quality of life questionnaire and World Health Organization Quality of Life) will be assessed.

**Results:**

This is the first trial to evaluate the effects of ACT on regulatory mechanisms of the cardiovascular system in PAD patients. If the results are as expected, they will provide evidence the ability of ACT to promote cardiovascular benefits in the symptomatic PAD population.

## Introduction

Peripheral artery disease (PAD) is the result of a chronic atherosclerotic process, which progressively leads to partial or total obstruction of the arteries that irrigate peripheral regions of the human body [1]. The main symptom of PAD is called intermittent claudication, which is characterized by pain, cramps, or burning that occurs in the lower limbs during physical activity and are relieved with rest [2].

Walking training (WT) is indicated as the initial and preferred form of clinical treatment for patients with symptomatic PAD [3], due to its benefits to functional capacity (e.g. increasing pain-free walking distance and total walking distance) [4], some parameters of cardiovascular health (e.g. lowering office blood pressure [BP], improving cardiac autonomic modulation, blood flow, and endothelial function), and quality of life [4–8]. Despite these benefits, WT usually implies in symptoms of pain in the lower limbs during execution, which could be considered as a barrier for long-term adherence to this modality of exercise program [9, 10]. Thus, identifying exercise modalities that have a positive effect on functional capacity, cardiovascular health, and quality of life and that, at the same time, minimize pain symptoms could be an interesting strategy for these patients.

Arm crank exercise training (ACT) has been applied as a non-painful modality strategy in patients with PAD with positive benefits on functional capacity and quality of life [11–13]. Regarding cardiovascular parameters, two previous studies demonstrated that ACT decreased office systolic BP in patients with symptomatic PAD [12, 13]. However, in these two studies, cardiovascular parameters were considered outcomes secondary and, therefore, they did not have an adequate study design, requiring further studies. In this context, the effects of ACT on other cardiovascular parameters (e.g. office and ambulatory BP, cardiac autonomic modulation, arterial stiffness, and vascular function), as well as on other parameters besides the cardiovascular system, such as cognitive function, which are impaired in these patients and have been related to the severity of the disease, are not known [14–17]. Thus, the aim of this study is to describe the protocol of a study protocol to analyze the effects of ACT on primary outcomes: office and ambulatory BP; and on secondary outcomes: BP determinants (i.e. heart rate variability, arterial stiffness and vascular function), functional capacity, cognition, and quality of life in patients with symptomatic PAD.

## Materials and methods

### Eligible participants

Patients with symptomatic PAD will be recruited from hospitals in São Paulo, Brazil. The inclusion criteria include: a) age > 40 years old; b) ankle brachial index (ABI) < 0.90 in one or both limbs; c) if the woman is in the post-menopausal period, without hormone replacement therapy; and d) able to perform physical exercise in upper and lower limbs. Exclusion criteria include: a) a change in medication during participation in the study; b) the presence of health problems that preclude performance of physical exercise during the participation in the study.

After agreeing to participate in the study, each patient will undergo a clinical evaluation. To confirm the PAD diagnosis, ABI will be assessed in accordance with previous guidelines [18]. Briefly, ABI will be measured as the highest systolic blood pressure in the posterior tibial or dorsalispedis artery, divided by the highest systolic blood pressure in the brachial artery. Blood pressure measurements will be recorded in both limbs using a doppler vascular monitor (DV160, Medmega, Brazil) and a sphygmomanometer.

To identify possible cardiovascular abnormalities during exercise, patients will be submitted to maximal treadmill test with a specific protocol for the PAD population [19]. The test will start at a constant speed of 3.2 km/h, with 2% incline increments every two minutes until exhaustion. During the test, heart rate will be continuously monitored by an electrocardiogram and BP will be obtained every two minutes using a mercury sphygmomanometer. Only patients with no restrictions to perform physical exercise training will be included.

### Study design

**Fig 1:**
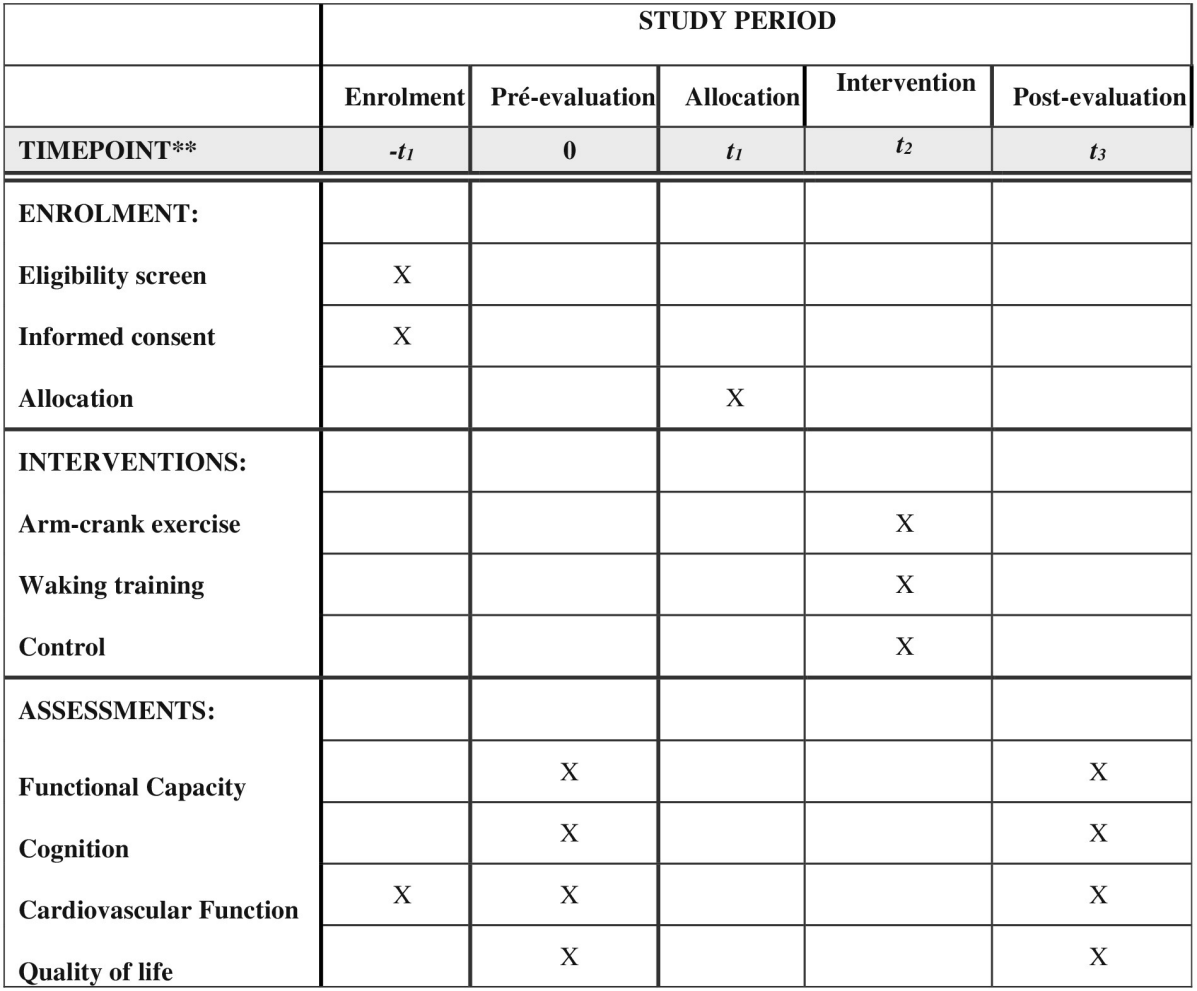
The recommendations for Interventional Trials (SPIRIT) schedule of enrollment, interventions and assessments.

This is a randomized clinical trial with single-blind data collection. The Recommendations for Interventional Trials (SPIRIT) flow chart and enrolment schedule, details of interventions, and assessments for the trial are given in Fig 1. The protocol study will be conducted according to the ethical principles governing research involving human subjects stipulated in Resolution 466/2012 of the Brazilian National Board of Health. The study protocol was approved by the Research Ethics Committee of Human Research of the Hospital Israelita Albert Einstein, Brazil (February 2020—CAAE: 81187317.6.0000.0071), Hospital das Clínicas, Faculty of Medicine, University of São Paulo, Brazil (March 2020—CAAE: 81187317.6.3002.0068),and registered and published in the ClinicalTrials.gov (registration number: NCT03837639). All participants will read and sign an informed consent form before enrollment. Participation will be voluntary, and all ethical principles of confidentiality and data protection will be maintained. The Research Ethics Committee of Human Research of the Hospital Israelita Albert Einstein will periodically monitor the study performance.

**Fig 2:**
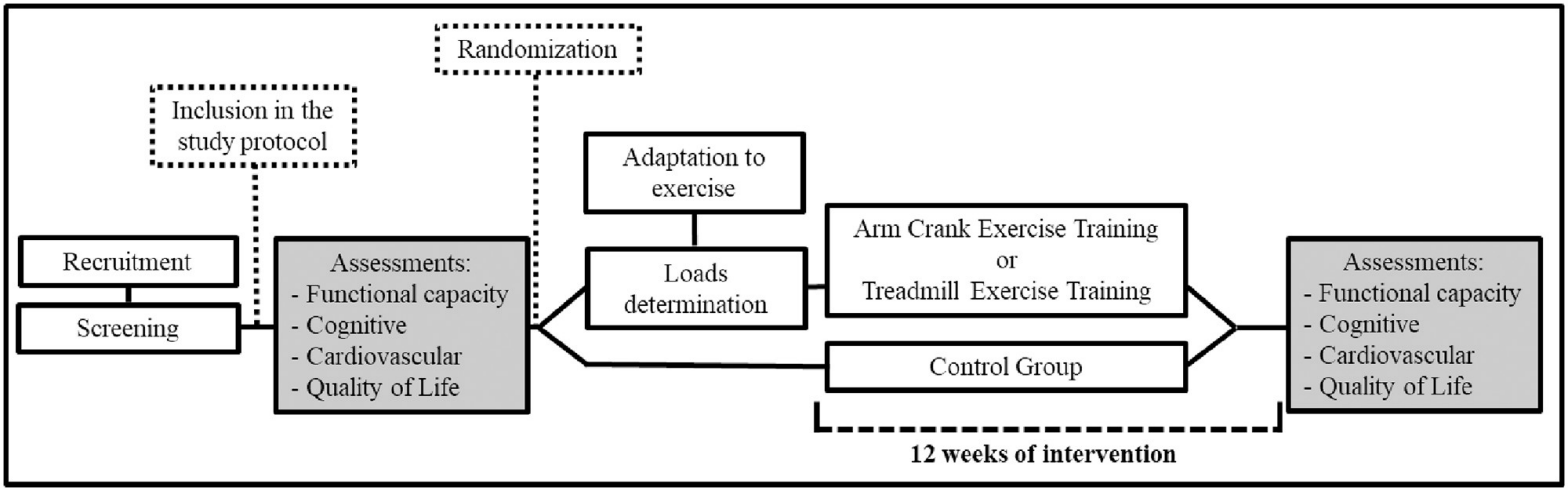
Design study.

Patients will perform two visits, with an interval of at least 48 hours. In the first visit, patients will perform the functional tests (i.e., 6-min walk test, 2 minute step test [2 MST], handgrip test, walking impairment questionnaire [WIQ], walking estimated limitation calculated by history [WELCH], Baltimore activity scale for intermittent claudication, and short physical performance battery [SPPB]), cognitive assessments, and answer the quality of life questionnaires (vascular quality of life questionnaire [VASCUQOL-6] and World Health Organization Quality of Life [WHOQOL brief]). On the second visit, patients will undergo cardiovascular assessments (i.e., office BP, ambulatory BP, central BP, heart rate variability, arterial stiffness and vascular function). Patients will then be randomly allocated in blocks to one of the three intervention groups (WT, ACT, and control groups [CG]) and will be re-evaluated after 12 weeks. The random allocation sequence will be generated by Researcher Randomizer (https://www.randomizer.org). These data will be collected by a trained kinesiologist who will be blind to the interventions. The study design is shown in Fig 2.

During the study, patients will be discontinued if they they choose to withdraw from the study or change their medication or present any health impairment that contraindicates the continuation of the practice of physical exercise during the study.

### Interventions—Arm crank exercise, treadmill exercise, and control group

Before starting the program, patients randomized to the ACT group will perform an adaptation session for familiarization with the ergometer and also to identify the training load. For this, patients will perform 10 bouts of two minutes of exercising with a minimum load and two minute passive intervals between bouts. Patients will be instructed to maintain 50 revolutions per minute (RPM) during the exercise. Subsequently, to determine the training load for each session, patients will perform a progressive test where the load will be increased by 10 watts every minute of the test, following the protocol of two minutes of exercise with two minutes of interval until the patient reports values of 13–15 on the subjective effort perception scale (Borg scale—6 to 20) [20].

Similarly, patients randomized to the WT group will perform 10 bouts of two minutes at 3.2 km/h, without incline, on the treadmill. Subsequently, the load will be progressively increased by 0.3 km/h every minute of the test until the patient reports values of 13–15 on the Borg scale. In both adaptations (ACT and WT), heart rate will be continuously monitored via a heart rate monitor (Polar A300, Polar, Finland) and the subjective effort perception scale (Borg scale—6 to 20) will be obtained at the end of each minute during the exercise.

Thus, during the first training sessions (i.e., ACT and WT), the workload corresponding to values of 13–15 on the Borg scale obtained in the progressive test will be employed. The Arm Crank exercise training and WT training will be performed twice a week for 12 weeks. The periodization of the training is shown in Table 1. In the first 3 weeks, each session will consist of 15 bouts of two minutes of exercise with two minutes of passive recovery. After that, the exercise time will be progressively increased by one minute every 3 weeks and the recovery period will be decreased, to a final maximum volume of 10 bouts of 5 minutes of exercise with 1 minute of passive recovery. The intensity of both exercise groups will be determined by the intensity equivalent to the range of 13–15 on the Borg scale (Borg scale—6 to 20 [20]. In addition, the intensity assessment will be performed during (each exercise bout) and at the end of each training session. All sessions will be supervised by a qualified kinesiologist with experience in vascular disease rehabilitation. Safety will be assessed by the proportion of participants who experience intervention-related adverse events during the study period.

**Table 1:** Periodization during the 12 weeks of training.

Weeks	Bouts	Active exercise	Total active time	Interval	Total interval time	Total session time
1–3 Week	15	2’	30’	2’	30’	60’
4–6 Week	14	3’	42’	1’30”	19’30”	62’
7–9 Week	12	4’	48’	1’	11’	59’
10–12 Week	10	5’	50’	1’	9’	59’

Patients randomized to the CG will also attend to meetings with the researcher team twice a week during the 12 weeks in order to minimize the effects of the patient’s bi-weekly commitment and displacement to the training site, to minimize the influence of the patient-researcher contact, and also minimize the convivial effect among the patients themselves, which will occur in the other two groups. During the meetings, patients will perform manual tasks with the use of artistic materials, cultural programs, cooking classes and home care, without any exercise component.

All patients included in the study will also receive recommendations to increase their levels of physical activity, which is the standard recommendation for clinical treatment for these patients [21].

### Measurements

For all measurements, patients will be instructed to eat a light meal 2 h beforehand, to avoid caffeinated beverages on the experimental days, and not to perform exercise in the previous 48 hours. With the exception of the six-minute walk test that will be performed along a 30-meter-long corridor, the other evaluations will be conducted at a laboratory, in a quiet environment, with monitored temperature, and no interruptions. The time of the day of the preintervention measurements of each patient will be maintained in the post-intervention evaluations.

#### Outcomes

The primary outcomes of the study are office and ambulatory BP. Secondary outcomes are: (1) cardiovascular function (central BP, heart rate variability, arterial stiffness, and vascular function); (2) functional capacity (six-minute walking test, 2 minute step test, handgrip test, Walking impairment questionnaire, Walking estimated limitation calculated by history, Baltimore activity scale for intermittent claudication, and short physical performance battery); (3) cognition (executive function and memory); and (4) quality of life (vascular quality of life questionnaire and World Health Organization Quality of Life).

### Primary outcome

#### Office blood pressure

Systolic and diastolic office BP will be measured by an automatic monitor (HEM-742, Omron Healthcare, Japan), during ten minutes at rest in a supine position. Three consecutive measurements will be performed with a one-minute interval between them, in both arms, and with the appropriate cuff size. The value used will be the average of the final two measures.

#### Ambulatory blood pressure

Ambulatory BP will be assessed by an oscillometric device (Dyna-MAPA, Cardios, Brazil) programmed to take measurements every 15 min for 24 hours. The recordings will only be accepted if at least 80% of the readings are successfully performed. Patients will be instructed to complete a diary to record the time of sleep, waking and daytime activities. Ambulatory data will be analysed using the averages of 24 h, awake, and asleep periods [22].

### Secondary outcomes

#### Cardiovascular function

*Central blood pressure*. Central BP will be obtained by the pulse wave analysis recorded in the left radial artery using applanation tonometry (SphygmoCor AtcorMedical, Sydney, Australia) and a validated transfer function algorithm provided by the Sphygmocor^®^ software will be use to estimate the central values of systolic, diastolic, and mean BP [23].

*Heart rate variability*. Heart rate variability will be assessed from the beat to beat intervals obtained using a heart rate monitor (V800, Polar Electro, Finland) for 10 min, with the patient in the supine position. Continuous stationary data recorded for at least 5 min will be used for analysis. All analyses will be performed using software (Kubios HRV, Biosignal Analysis and Medical Imaging Group, Finland) according to the recommendations of the Task Force for heart rate variability [24]. The time-domain parameters (SDNN—standard deviation of all RR intervals, RMSSD—root mean square of the squared differences between adjacent normal RR intervals, pNN50 –the percentage of adjacent intervals over 50ms) and frequency-domain (low frequency, high frequency, and low frequency/high frequency) parameters will be analyzed as previously described [24].

*Arterial stiffness*. The arterial stiffness parameters, such as pulse pressure (difference between systolic and diastolic blood pressure) and augmentation index (the proportion of pulse pressure that is attributed to the reflected pulse wave) will be obtained through applanation tonometry (SphygmoCor, AtCor Medical, Australia), in the radial artery. Carotid-femoral pulse wave velocity will be measured by applanation tonometry (Sphygmocor, AtCor Medical, Australia) following the guidelines of the Clinical Application of Arterial Stiffness, Task Force III [23]. The distance from the carotid artery to the suprasternal notch and the femoral artery to the suprasternal notch will be measured using a standard tape. Electrocardiogram will be simultaneously assessed to obtain heart rate and, according to a “foot-to-foot” method, the time difference between the points will be measured. The distance between the two arteries will then be divided by the time difference.

*Vascular function*. Vascular function will be estimated by resting blood flow and flow-mediated dilation measurements obtained by an ultrasound technique according to recent recommendations [25]. Images of the brachial arteries will be recorded by a two-dimensional ultrasound with a spectral Doppler and linear transducer (Ultra-0122, Philips, The Netherlands). For this, each patient will remain in the supine position for at least 20 minutes. After location of the arteries, the transducer will be positioned and to attest to the good quality of the arterial pulse obtained, the Doppler sound will be activated.

The contrast resolution, depth, and gain will be adjusted to optimize the longitudinal images of the lumen/arterial wall interface. Insonation angle, corrected at 60°, blood velocity spectra will be simultaneously recorded via the pulsed-wave mode at linear frequencies of 13 and 6.0 MHz, respectively.

Baseline diameter and blood velocity waveforms will be continuously recorded over 120 s. After that, a cuff, placed distal to the image capture, will be inflated with a pressure above 50 mm Hg of the systolic BP measured before the examination. The image and Doppler recordings will be resumed 30 s before deflation and will be maintained for 180s after deflation.

The diameters and post-occlusion blood flow velocities will be measured after the release. The vasodilatory capacity will be calculated by the flow-mediated dilation, the percentage increase in diameter of the brachial artery post occlusion compared to their baseline values.

#### Functional capacity

*Six-minute walk test*. The six-minute walk test will be performed along a 30-meter-long corridor, as previously described [26, 27]. Briefly, patients will be encouraged to “walk at their usual pace for six-minutes and cover as much ground as possible” and rest if necessary. The outcomes will be the onset claudication distance (distance walked when the patients related the occurrence of symptoms of intermittent claudication) and six-minute total walking distance (maximum distance achieved by the patient at the end of the test).

*Two-minute step test*. The two-minutes step test will be performed as previously described [28]. Patients will be required to walk on the spot for 2 minutes, and the maximum number of steps will be counted. The walk will consist of alternate elevation of the knees to the mean height of the thigh (midpoint between the patella and the anterior superior iliac spine). Patients will be instructed to complete as many steps as possible during the test.

*Walking impairment questionnaire*. The walking impairment questionnaire [29] contains three domains measuring three factors of walking impairment: walking distance, walking speed, and the ability to climb stairs. Patients will be asked how difficult it was to walk in these situations and will be required to answer from among the options “none, slight, some, much, or unable”. Each domain is scored on a 0 to 100 scale, where 0 represents extreme limitation and 100 represents no difficulties walking long distances, walking rapidly, or climbing 3 flights of stairs, respectively.

*Walking estimated limitation calculated by history*. The walking estimated-limitation calculated by history is a four-question questionnaire, in which the first 3 questions are related to how long patients can perform the task easily on level ground and without stopping at different walking speeds and the last question is related to speed comparisons with their relatives, friends, or people of the same age. The score (ranges from 0 to 100) is calculated as the sum of the values for the first three, minus one, multiplied by the coefficient for the final (walking speed) questionnaire item [30].

*Handgrip test*. The handgrip test will be performed to evaluate the handgrip strength. The test will be performed using a dynamometer with digital display (EH101, Camry, USA), calibrated with a scale from 0 to 100 kgf, following a previous protocol [31]. Patients will be evaluated seated with a slightly adducted shoulder, elbow flexed at 90°, forearm and wrist in neutral position. Three attempts at the test will be performed in each of the dominant and non-dominant hands, alternately, and the highest value will be used for analysis.

*Baltimore activity scale for intermittent claudication*. The Baltimore Activity Scale for Intermittent Claudication will be obtained following a previous protocol [32]. The scale consists of five questions related to the symptoms of intermittent claudication. For each question, the patient selects the answer that best describes their symptoms and level of physical activity. Values range from 0 to 2 points, and the total score is the sum of the points from the 5 questions. The score ranges from 0 to 10, with zero being the lowest level of physical activity and ten being the highest [32].

*Short physical performance battery*. Functional capacity will also be obtained by the short physical performance battery, as previously described [33]. The short physical performance battery is a group of measures that combines the results of gait speed, chair stand, and balance tests. The total score will be calculated from the performance in the three tests, ranging from 0 to 12, with 0 representing the worst function and 12 the best function.

#### Cognition

Standardized cognitive tasks to quantify executive function and memory will be evaluated as previously described [34]. These assessments will be carried out on paper and include: a) executive function—Test A and B, coding of digit symbols; b) memory: Hopkins verbal learning test (immediate and delayed recovery), forward and backward digit range; c) verbal fluency: task of generating words ("S" and animals).

#### Quality of life

*Vascular quality of life questionnaire– 6*. The quality of life will be evaluated by the vascular quality of life questionnaire VascuQoL-6, as previously described [35]. The questionnaire is composed of six items to evaluate the impact of disease on social aspects and the capacity to perform daily activities. Each item is scored from 1–4. The total score is achieved by summarizing the score on each item, resulting in a score between 6 and 24. A higher value indicates better health status.

*World Health Organization Quality of Life (WHOQOL) brief*. The WHOQOL-brief instrument measures different aspects of physical and mental health, which include the following dimensions: general health status, functional capacity, physical aspects, pain, vitality, mental health, emotional aspects, and social aspects of life. Each answer receives a score, which is added to a constant to determine the different components of quality of life [36].

### Intervening variables

In order to minimize the possible influences of intervening variables, and to ensure that the changes generated in the outcomes are caused by ACT or WT interventions, medications, dietary pattern, and physical activity levels will be monitored.

To control medication, each week the researcher will be responsible for filling a container with the medications prescribed for the patient, according to the frequency, time, and recommended dosage. At the end of the week (in the second weekly session), the researcher will check if the patient correctly took the medications. Patients will be followed during the 12 week period of the intervention.

For food monitoring, before and after the intervention period, patients will be asked to complete a food diary for four days of the week, including at least one day of the weekend. Based on this information, it will be possible to analyze changes in the dietary patterns of the patients, as well as to estimate the caloric intake ingested before and after the intervention period.

Finally, patients will use a smartwatch (A300, Polar, Finland) to estimate their physical activity levels. The POLAR A300 is a monitor coupled with a 3D accelerometer that records patient’s movements. With the watch, it is possible to analyze the frequency, intensity, and regularity of the movements and, consequently, to determine physical activity levels. Patients will use this monitor for one week before the first evaluation and one week after the final week of training, to allowing identification of possible changes in physical activity level after the ACT, WT, and CG interventions.

### Statistical analysis

#### Power and sample size

The sample size was determined by the a priori specific sample calculation for clinical trials involving parallel groups, in this case two-way ANOVA with repeated measures, suggested by Beck (2013) [37]. Using GPower 3.1.9.2 software it was considered an effect size of 0.25; α of 0.05; power (1—β) of 0.80; correlation coefficient between repeated measures of 0.6; non-spherical correction (ɛ) of 1 and three groups two measures. Thus, the minimum total size reached 36 subjects (12 per group) with 83% power. Considering the possibility of sample loss, the sample was inflated by 20%, resulting in a sample of 45 patients (15 per group).

#### Analysis plan

The normality and homogeneity of variance will be performed using the Shapiro-Wilks and Levene test. For pre-intervention comparisons, we will use one-way ANOVA or Qui-square. For the pre and post comparison, we will use ANOVA for repeated measures, establishing as the main factors: intervention (WT, ACT and control) and time (pre- and post-intervention). Newman-Keuls post-hoc test will be used with P <0.05 value as significant. Intention-to-treat will be used for the patients with incomplete follow-period.

### Trial status

Enrollment of patients started in December 2019. Collection was suspended between March 2020 and July 2021 because of the COVID-19 pandemic. Thirty percent of the data collection has already been completed. Recruitment is scheduled to be completed on 31.11.2022.

## Discussion

Previous studies [13, 38] comparing ACT versus WT have shown similar improvement in the pain-free walking distance and total walking distance during the treadmill exercise test. However, the effects of ACT on the walking capacity in other conditions (e.g., walking fast, climbing stairs, the six-minute walking test, etc), as well as on the other parameters of functional capacity, such as strength and balance, are still unclear. This is relevant, since these patients also present alterations in different parameters of functional capacity [10, 27, 29, 39].

Regarding cardiovascular parameters, two previous studies [12, 13] demonstrated the benefits of ACT on systolic BP compared to the control group. We expect to confirm these results, and expand the analysis to other cardiovascular parameters, including ambulatory BP, arterial stiffness, cardiac autonomic regulation, and vascular function. Due to the systemic effects of exercise training for upper limbs on cardiovascular function [40], we expect similar results with ACT to those observed in WT, which includes improvements in cardiac autonomic modulation [7], vascular function [7], and ambulatory blood pressure variability [5].

Patients with symptomatic PAD usually present impairments in cognitive function [15] however, the effect of exercise training on the cognitive function of these patients is still unclear. In other populations without PAD (e.g. older people, etc.) exercise training has been shown to improve cognitive function [41–45]. Several mechanisms have been proposed to explain the effects of exercise training on cognitive function, including improvements in vascular function of cerebral arteries [46]. In this context, as patients with PAD commonly present impairments in cerebral vascular functions [46, 47], it is possible that the systemic cardiovascular effects of ACT can improve cognitive functions.

Patients with PAD have limited exercise capacity, reduced functional performance, and poor cardiorespiratory fitness, as well as several comorbidities that can lead to a significant impairment in health-related quality of life [48]. Thus, full understanding of the health benefits resulting from exercise training must include outcomes related to quality of life [49]. Since both modalities employed in this study can significantly improve physical and cardiovascular function, we expect that these aspects may help to alleviate the burden of the disease (improving symptoms and reducing comorbidities related to PAD), which could have a strong bearing on perceived health-related quality of life.

In summary, the present study aims to provide a broader analysis of the effects of ACT in patients with symptomatic PAD. The current study might reinforce and expand the use of ACT as a treatment strategy for patients with PAD and intermittent claudication.

## Supporting information

S1 Checklist SPIRIT 2013 checklist: Recommended items to address in a clinical trial protocol and related documents*. (DOC)

S1 File Ethical approval document. (PDF)

S2 File Translation ethics. (DOCX)

S3 File Ethical approval document. (PDF)

S4 File Translation ethics. (DOCX)

S5 File Study protocol approved. (DOCX)

S6 File Translation of the study protocol approved. (DOCX)

S7 File Questionnaire on inclusivity in global research. (DOCX)

## References

[1] Bradberry JC. Peripheral arterial disease: pathophysiology, risk factors, and role of antithrombotic therapy. J Am Pharm Assoc. 2004; 44(2 Suppl 1): S37–44. doi: 10.1331/154434504322904596 15095934

[2] Wolosker N, Rosoky RA, Nakano L, Basyches M, Puech-Leão P. Predictive value of the ankle-brachial index in the evaluation of intermittent claudication. Fac Med São Paulo. 2000; 55(2): 61–64. doi: 10.1590/s0041-87812000000200005 10959125

[3] Rooke TW, Hirsch AT, Misra S, Sidawy AN, Beckman JA, Findeiss L, et al. Management of patients with peripheral artery disease (compilation of 2005 and 2011 ACCF/AHA guideline recommendations): a report of the American College of Cardiology Foundation/American Heart Association Task Force on Practice Guidelines. Circulation. 2013; 127(13): 1425–1443. doi: 10.1161/CIR.0b013e31828b82aa 23457117

[4] Gardner AW. Exercise rehabilitation for peripheral artery disease: An exercise physiology perspective with special emphasis on the emerging trend of home-based exercise. VASA. 2015; 44(6): 405–417. doi: 10.1024/0301-1526/a000464 26515218

[5] Grizzo Cucato G, de Moraes Forjaz CL, Kanegusuku H, da Rocha Chehuen M, Riani Costa LA, Wolosker N, et al. Effects of walking and strength training on resting and exercise cardiovascular responses in patients with intermittent claudication. VASA. 2011; 40(5): 390–397. doi: 10.1024/0301-1526/a000136 21948782

[6] Januszek R, Mika P, Konik A, Petriczek T, Nowobilski R, Nizankowski R. Effect of treadmill training on endothelial function and walking abilities in patients with peripheral arterial disease. J Cardiol. 2014; 64(2): 145–151. doi: 10.1016/j.jjcc.2013.12.002 24438856

[7] Chehuen M, Cucato GG, Carvalho CRF, Ritti-Dias RM, Wolosker N, Leicht AS, et al. Walking training at the heart rate of pain threshold improves cardiovascular function and autonomic regulation in intermittent claudication: A randomized controlled trial. J Sci Med Sport. 2017; 20(10): 886–892. doi: 10.1016/j.jsams.2017.02.011 28389218

[8] Andreozzi GM, Leone A, Laudani R, Deinite G, Martini R. Acute impairment of the endothelial function by maximal treadmill exercise in patients with intermittent claudication, and its improvement after supervised physical training. Int Angiol. 2007; 26 (1): 12–17. 17353883

[9] Barbosa JP, Farah BQ, Chehuen M, Cucato GG, Farias JC, Wolosker N, et al. Barriers to physical activity in patients with intermittent claudication. Int J Behav Med. 2015; 22 (1): 70–76. doi: 10.1007/s12529-014-9408-4 24715636

[10] Cavalcante BR, Farah BQ, Barbosa JP, Cucato GG, Chehuen MR, Santana FD, et al. Are the barriers for physical activity practice equal for all peripheral artery disease patients?. Arch Phys Med Rehabil. 2015; 96(2): 248–252. doi: 10.1016/j.apmr.2014.09.009 25281872

[11] Walker RD, Nawaz S, Wilkinson CH, Saxton JM, Pockley AG, Wood RF. Influence of upper- and lower-limb exercise training on cardiovascular function and walking distances in patients with intermittent claudication. J Vasc Surg. 2000; 31(4):662–9. doi: 10.1067/mva.2000.104104 10753273

[12] Zwierska I, Walker RD, Choksy SA, Male JS, Pockley AG, Saxton JM. Upper- vs lower-limb aerobic exercise rehabilitation in patients with symptomatic peripheral arterial disease: a randomized controlled trial. J Vasc Surg. 2005; 42(6): 1122–1130. doi: 10.1016/j.jvs.2005.08.021 16376202

[13] Treat-Jacobson D, Bronas UG, Leon AS. Efficacy of arm-ergometry versus treadmill exercise training to improve walking distance in patients with claudication. Vasc Med. 2009; 14(3): 203–213. doi: 10.1177/1358863X08101858 19651669

[14] Kanegusuku H, Cucato GG, Domiciano RM, Longano P, Puech-Leao P, wolosker N, et al. Impact of obesity on walking capacity and cardiovascular parameters in patients with peripheral artery disease: A cross-sectional study. J Vasc Nurs. 2020; 38(2): 66–71. doi: 10.1016/j.jvn.2020.02.004 32534655

[15] Cavalcante BR, Germano-Soares AH, Gerage AM, Leicht A, Tassitano RM, Bortolotti H, et al. Association between physical activity and walking capacity with cognitive function in peripheral artery disease patients. Eur J Vasc Endovasc Surg. 2018; 55(5): 672–678. doi: 10.1016/j.ejvs.2018.02.010 29580833

[16] Lima AHRA, Chehuen M, Cucato GG, Soares H, Askew CD, Barbosa JPAS, et al. Relationship between walking capacity and ambulatory blood pressure in patients with intermittent claudication. Blood Press Monit. 2017; 22(3): 115–121. doi: 10.1097/MBP.0000000000000243 28195842

[17] Lima A, Soares AHG, Cucato GG, Leicht AS, Franco FGM, Wolosker N, et al. Walking Capacity Is Positively Related with Heart Rate Variability in Symptomatic Peripheral Artery Disease. Eur J Vasc Endovasc Surg. 2016; 52(1): 82–89. doi: 10.1016/j.ejvs.2016.03.029 27161329

[18] Aboyans V, Ricco JB, Bartelink MEL, Bjorck M, Brodmann M, Cohnert T, et al. 2017 ESC Guidelines on the Diagnosis and Treatment of Peripheral Arterial Diseases, in collaboration with the European Society for Vascular Surgery (ESVS): Document covering atherosclerotic disease of extracranial carotid and vertebral, mesenteric, renal, upper and lower extremity arteriesEndorsed by: the European Stroke Organization (ESO) The Task Force for the Diagnosis and Treatment of Peripheral Arterial Diseases of the European Society of Cardiology (ESC) and of the European Society for Vascular Surgery (ESVS). Eur Heart J. 2018; 39(9): 763–816. doi: 10.1093/eurheartj/ehx095 28886620

[19] Gardner AW, Skinner JS, Cantwell BW, Smith LK. Progressive vs single-stage treadmill tests for evaluation of claudication. Med Sci Sports Exerc. 1991; 23(4): 402–428. 2056896

[20] Borg GAV. Psychophysical bases of perceived exertion. Med Sci Sports Exerc. 1982; 14(5): 377–381. 7154893

[21] Brook RD, Appel LJ, Rubenfire M, Ogedegbe G, Bisognano JD, Elliott WJ, et al. Beyond medications and diet: alternative approaches to lowering blood pressure: a scientific statement from the american heart association. Hypertension. 2013; 61(6): 1360–83. doi: 10.1161/HYP.0b013e318293645f 23608661

[22] Parati G, Stergiou G, O’Brien E, Asmar R, Beilin L, Bilo G, et al. European Society of Hypertension practice guidelines for ambulatory blood pressure monitoring. J Hypertens. 2014; 32(7): 1359–1366. doi: 10.1097/HJH.0000000000000221 24886823

[23] Van Bortel LM, Duprez D, Starmans-Kool MJ, Safar ME, Giannattasio C, Cockcroft J, et al. Task Force III: recommendations for user procedures. Am J Hypertens. 2002; 15(5): 445–452. doi: 10.1016/s0895-7061(01)02326-3 12022247

[24] Task Force of the European Society of Cardiology, The North American Society of Pacing and Electrophysiology. Heart rate variability: standards of measurement, physiological interpretation and clinical use. Task Force of the European Society of Cardiology and the North American Society of Pacing and Electrophysiology. Circulation. 1996; 93(5): 1043–1065. 8598068

[25] Thijssen DH, Black MA, Pyke KE, Padilla J, Atkinson G, Harris RA, et al. Assessment of flow-mediated dilation in humans: a methodological and physiological guideline. Am J Physiol Heart Circ Physiol. 2011; 300(1): H2–12. doi: 10.1152/ajpheart.00471.2010 20952670 PMC3023245

[26] Montgomery PS, Gardner AW. The clinical utility of a six-minute walk test in peripheral arterial occlusive disease patients. J Am Geriatr Soc. 1998; 46(6): 706–711. doi: 10.1111/j.1532-5415.1998.tb03804.x 9625185

[27] Ritti-Dias RM, Sant’anna FDS, Braghieri HA, Wolosker N, Puech-Leao P, Lanza FC, et al. Expanding the use of six-minute walking test in patients with intermittent claudication. Ann Vasc Surg. 2021; 70: 258–262. doi: 10.1016/j.avsg.2020.07.047 32800882

[28] Braghieri HA, Kanegusuku H, Corso SD, Cucato GG, Monteiro F, Wolosker N, et al. Validity and reliability of 2-min step test in patients with symptomatic peripheral artery disease. J Vasc Nurs. 2021; 39(2): 33–38. doi: 10.1016/j.jvn.2021.02.004 34120695

[29] Ritti-Dias RM, Gobbo LA, Cucato GG, Wolosker N, Jacob Filho W, Santarém JM, et al. Translation and validation of the walking impairment questionnaire in Brazilian subjects with intermittent claudication. Arq Bras Cardiol. 2009; 92(2): 136–149. doi: 10.1590/s0066-782x2009000200011 19360247

[30] Cucato GG, Correia MdA, Farah BQ, Saes GF, Lima AHdA, Ritti-Dias RM, et al. Validation of a brazilian portuguese version of the walking estimated-limitation calculated by history (WELCH). Arq Bras Cardiol. 2016; 106(1): 49–55. doi: 10.5935/abc.20160004 26647720 PMC4728595

[31] Silva GO, Farah BQ, Germano-Soares AH, Andrade-Lima A, Santana FS, Rodrigues SL, et al. Acute blood pressure responses after different isometric handgrip protocols in hypertensive patients. Clinics. 2018; 73: e373. doi: 10.6061/clinics/2018/e373 30365821 PMC6172980

[32] Gardner AW, Montgomery PS. The baltimore activity scale for intermittent claudication: a validation study. Vasc Endovascular Surg. 2006; 40(5): 383–391. doi: 10.1177/1538574406288575 17038572

[33] Guralnik JM, Simonsick EM, Ferrucci L, Glynn RJ, Berkman LF, Blazer DG, et al. A short physical performance battery assessing lower extremity function: association with self-reported disability and prediction of mortality and nursing home admission. J Gerontol. 1994; 49(2): M85–94. doi: 10.1093/geronj/49.2.m85 8126356

[34] Carson N, Leach L, Murphy KJ. A re-examination of Montreal Cognitive Assessment (MoCA) cutoff scores. Int J Geriatr Psychiatry. 2018; 33(2): 379–388. doi: 10.1002/gps.4756 28731508

[35] de Almeida Correia M, Andrade-Lima A, Mesquita de Oliveira PL, Domiciano RM, Ribeiro Domingues WJ, Wolosker N, et al. Translation and validation of the brazilian-portuguese short version of vascular quality of life questionnaire in peripheral artery disease patients with intermittent claudication symptoms. Ann Vasc Surg. 2018; 51(1): 48–54. doi: 10.1016/j.avsg.2018.02.026 29772330

[36] The World Health Organization quality of life assessment (WHOQOL): Position paper from the World Health Organization. Soc Sci Med. 1995; 41(10): 1403–1409. doi: 10.1016/0277-9536(95)00112-k 8560308

[37] Beck TW. The importance of a priori sample size estimation in strength and conditioning research. J Strength Cond Res. 2013; 27(8): 2323–2337. doi: 10.1519/JSC.0b013e318278eea0 23880657

[38] Bronas UG, Treat-Jacobson D, Leon AS. Comparison of the effect of upper body-ergometry aerobic training vs treadmill training on central cardiorespiratory improvement and walking distance in patients with claudication. J Vasc Surg. 2011; 53(6): 1557–1564. doi: 10.1016/j.jvs.2011.01.077 21515017

[39] Longano P, Kanegusuku H, Correia MA, Puech-Leão P, Wolosker N, Cucato GG, et al. Are cardiovascular function and habitual physical activity levels similar in patients with classic and atypical claudication symptoms? A cross-sectional study. Vascular. 2020; 28(4): 360–367. doi: 10.1177/1708538120911292 32212916

[40] Ritti-Dias RM, Correia MA, Andrade-Lima A, Cucato GG. Exercise as a therapeutic approach to improve blood pressure in patients with peripheral arterial disease: current literature and future directions. Expert Rev Cardiovasc Ther. 2019; 17(1): 65–73. doi: 10.1080/14779072.2019.1553676 30481076

[41] Yang SY, Shan CL, Qing H, Wang W, Zhu Y, Yin MM, et al. The effects of aerobic exercise on cognitive function of alzheimer’s disease patients. CNS Neurol Disord Drug Targets. 2015; 14(10): 1292–1297. doi: 10.2174/1871527315666151111123319 26556080

[42] Okamura H, Otani M, Shimoyama N, Fujii T. Combined exercise and cognitive training system for dementia patients: a randomized controlled trial. Dement Geriatri Cogn Disord. 2018; 45(5–6): 318–325. doi: 10.1159/000490613 30036871

[43] Bossers WJ, van der Woude LH, Boersma F, Hortobagyi T, Scherder EJ, van Heuvelen MJ. A 9-week aerobic and strength training program improves cognitive and motor function in patients with dementia: a randomized, controlled trial. Am J Geriatr Psychiatry. 2015; 23(11): 1106–1116. doi: 10.1016/j.jagp.2014.12.191 25648055

[44] Song D, Yu DSF. Effects of a moderate-intensity aerobic exercise programme on the cognitive function and quality of life of community-dwelling elderly people with mild cognitive impairment: A randomised controlled trial. Int J Nurs Stud. 2019; 93: 97–105. doi: 10.1016/j.ijnurstu.2019.02.019 30901716

[45] Hsu CL, Best JR, Davis JC, Nagamatsu LS, Wang S, Boyd LA, et al. Aerobic exercise promotes executive functions and impacts functional neural activity among older adults with vascular cognitive impairment. Br J Sports Med. 2018; 52(3): 184–191. doi: 10.1136/bjsports-2016-096846 28432077

[46] Leardini-Tristao M, Charles AL, Lejay A, Pizzimenti M, Meyer A, Estato V, et al. Beneficial effect of exercise on cognitive function during peripheral arterial disease: potential involvement of myokines and microglial anti-inflammatory phenotype enhancement. J Clin Med. 2019; 8(5): 653. doi: 10.3390/jcm8050653 31083472 PMC6571759

[47] Waldstein SR, Tankard CF, Maier KJ, Pelletier JR, Snow J, Gardner AW, et al. Peripheral arterial disease and cognitive function. Psychosom Med. 2003; 65(5): 757–763. doi: 10.1097/01.psy.0000088581.09495.5e 14508017

[48] Spronk S, White JV, Bosch JL, Hunink MG. Impact of claudication and its treatment on quality of life. Semin Vasc Surg. 2007; 20(1): 3–9. doi: 10.1053/j.semvascsurg.2007.02.003 17386358

[49] Guidon M, McGee H. One-year effect of a supervised exercise programme on functional capacity and quality of life in peripheral arterial disease. Disabil Rehabil. 2013; 35(5): 397–404. doi: 10.3109/09638288.2012.694963 22804715

