# Supplementary material for: Effects of arm-crank exercise on cardiovascular function, functional capacity, cognition and quality of life in patients with peripheral artery disease: Study protocol for a randomized controlled trial
Source: PLoS One. 2022 May 5;17(5):e0267849. doi: 10.1371/journal.pone.0267849 (PMC9070866; doi:10.1371/journal.pone.0267849)
Supplement: S1 File — (PDF) [file pone.0267849.s002.pdf]

PARECER CONSUBSTANCIADO DO CEP

DADOS DO PROJETO DE PESQUISA

Título da Pesquisa: Efeitos agudos e crônicos do exercício físico realizado em ergômetro de braço na função e regulação cardiovascular, capacidade funcional, cognitiva e qualidade de vida de pacientes com doença arterial periférica

Pesquisador: NELSON WOLOSKER

Área Temática:

Versão: 6

CAAE: 81187317.6.0000.0071

Instituição Proponente: SOCIEDADE BENEF ISRAELITABRAS HOSPITAL ALBERT EINSTEIN

Patrocinador Principal: Financiamento Próprio

DADOS DA NOTIFICAÇÃO

Tipo de Notificação: Outros

Detalhe: Retificação do parecer emitido em 06/02/2020

Justificativa: A presente notificação trata-se de uma retificação quanto ao parecer emitido ao

Data do Envio: 11/02/2020

Situação da Notificação: Parecer Consubstanciado Emitido

DADOS DO PARECER

Número do Parecer: 3.834.172

Apresentação da Notificação:

Envio de errata para correção e ratificação de TCLE aprovado referente ao Projeto: "Efeitos agudos e crônicos do exercício físico realizado em ergômetro de braço na função e regulação cardiovascular, capacidade funcional, cognitiva e qualidade de vida de pacientes com doença arterial periférica".

Objetivo da Notificação:

Notificar erro encontrado em relação a data e versão emitida em parecer consubstanciado.

Avaliação dos Riscos e Benefícios:

Riscos e benefícios já avaliados e mantidos.

Comentários e Considerações sobre a Notificação:

Pesquisador envia notificação para registro de errata ao parecer nº3.826.253, com os seguintes dizeres: Solicitamos emissão de errata ao parecer nº 3.826.253 emitido em 06/02/2020, a respeito da versão dos TCLES apresentados para análise. Gostaríamos que fosse registrada a data correta da versão do TCLE para crônico, ratificando:
1-Termo de Consentimento Livre e Esclarecido (agudo) – Versão 3 datada de 21 de Dezembro de 2019.
2-Termo de Consentimento Livre e Esclarecido (crônico) – Versão 3 datada de 21 de Dezembro de 2019.

Após análise não foram encontrados impedimentos e a correção ao parecer foi aprovada.

Considerações sobre os Termos de apresentação obrigatória:

Termos em acordo com as Resoluções vigentes.

Recomendações:

Sem recomendações adicionais.

Conclusões ou Pendências e Lista de Inadequações:

Após análise, foi emitida errata ao parecer e os Termos de consentimento em suas respectivas versões foram aprovados:
1-Termo de Consentimento Livre e Esclarecido (agudo) – Versão 3 datada de 21 de Dezembro de 2019.
2-Termo de Consentimento Livre e Esclarecido (crônico) – Versão 3 datada de 21 de Dezembro de 2019

Considerações Finais a critério do CEP:

NOTIFICAÇÃO APROVADA PELO CEP DO HOSPITAL ISRAELITA ALBERT EINSTEIN EM AD REFERENDUM.

Este parecer foi elaborado baseado nos documentos abaixo relacionados:

Tipo Documento	Arquivo	Postagem	Autor	Situação
Outros	TCLE_cronico_limpo.docx	11/02/2020 11:33:39	NELSON WOLOSKER	Postado
Outros	TCLE_cronico.docx	11/02/2020	NELSON	Postado

Endereço: Av. Albert Einstein 627 - 2ss
Bairro: Morumbi **CEP:** 05.652-000
UF: SP **Município:** SAO PAULO
Telefone: (11)2151-3729 **Fax:** (11)2151-0273 **E-mail:** cep@einstein.br

Outros	TCLE_cronico.docx	11:33:45	NELSON	Postado
Outros	Correcao_data_TCLE_Cronico.pdf	11/02/2020 11:40:03	NELSON WOLOSKER	Postado
Outros	Notificacao_errata_NW.doc	12/02/2020 12:30:13	Fabio Pires de Souza Santos	Aceito

Situação do Parecer:
Aprovado

Necessita Apreciação da CONEP:
Não

SAO PAULO, 12 de Fevereiro de 2020

Assinado por:
Fabio Pires de Souza Santos
(Coordenador(a))

Endereço: Av. Albert Einstein 627 - 2ss
Bairro: Morumbi **CEP:** 05.652-000
UF: SP **Município:** SAO PAULO
Telefone: (11)2151-3729 **Fax:** (11)2151-0273 **E-mail:** cep@einstein.br
