# Supplementary material for: Effects of arm-crank exercise on cardiovascular function, functional capacity, cognition and quality of life in patients with peripheral artery disease: Study protocol for a randomized controlled trial
Source: PLoS One. 2022 May 5;17(5):e0267849. doi: 10.1371/journal.pone.0267849 (PMC9070866; doi:10.1371/journal.pone.0267849)
Supplement: S2 File — (DOCX) [file pone.0267849.s003.docx]

Research Title: Acute and chronic effects of physical exercise performed on an arm ergometer on cardiovascular function and regulation, functional, cognitive capacity and quality of life of patients with peripheral arterial disease

Researcher: NELSON WOLOSKER

Thematic Area:

Version: 6

CAAE: 81187317.6.0000.0071

Proponent Institution: SOCIEDADE BENEF ISRAELITA BRAS HOSPITAL ALBERT EINSTEIN

Main Sponsor: Own Financing

NOTIFICATION DATA

Notification Type: Other

Detail: Correction of the opinion issued on 02/06/2020

Justification: This notification is a correction to the opinion issued on the Date of Submission: 11/02/2020

Notification Status: Consubstantiated Opinion Issued

OPINION DATA

Opinion Number: 3.834.172

Presentation of the Notification:

Submission of errata for correction and ratification of the ICF approved for the Project: "Acute and chronic effects of physical exercise performed on an arm ergometer on cardiovascular function and regulation, functional, cognitive capacity and quality of life of patients with peripheral arterial disease".

Purpose of Notification:

Notify error found in relation to the date and version issued in a substantiated opinion.

Assessment of Risks and Benefits:

Risks and benefits already evaluated and maintained.

Notification Comments and Considerations:

Researcher sends notification for registration of errata to Opinion No. 3.826.253 with the following wording: We request the issuance of errata to Opinion No. 3.826.253 issued on 2/6/2020. regarding the version of the TCLES submitted for analysis. We would like to have the correct date of the version of the informed consent form recorded for chronic. confirming:

1-Free and Informed Consent Term (acute) - Version 3 dated December 21. 2019.

2 -Free and Informed Consent Term (chronic) - Version 3 dated December 21. 2019.

After analysis. no impediments were found and the correction to the opinion was approved.
