# Supplementary material for: Effects of arm-crank exercise on cardiovascular function, functional capacity, cognition and quality of life in patients with peripheral artery disease: Study protocol for a randomized controlled trial
Source: PLoS One. 2022 May 5;17(5):e0267849. doi: 10.1371/journal.pone.0267849 (PMC9070866; doi:10.1371/journal.pone.0267849)
Supplement: S3 File — (PDF) [file pone.0267849.s004.pdf]

PARECER CONSUBSTANCIADO DO CEP

Elaborado pela Instituição Coparticipante

DADOS DO PROJETO DE PESQUISA

Título da Pesquisa: Efeitos agudos e crônicos do exercício físico realizado em ergômetro de braço na função e regulação cardiovascular, capacidade funcional, cognitiva e qualidade de vida de pacientes com doença arterial periférica

Pesquisador: NELSON WOLOSKER

Área Temática:

Versão: 1

CAAE: 81187317.6.3002.0068

Instituição Proponente: Hospital das Clínicas da Faculdade de Medicina da USP

Patrocinador Principal: Financiamento Próprio

DADOS DO PARECER

Número do Parecer: 3.912.388

Apresentação do Projeto:

Pacientes com doença arterial periférica tem alto risco de eventos cardiovasculares fatais e não fatais. Estudos com treinamento em ergômetro de braço (EB) têm demonstrado trazer benefícios na capacidade funcional nesses pacientes contornando a principal barreira para a prática de exercício nesses pacientes, a dor. Entretanto, o efeito desse tipo de treinamento em indicadores da função cardiovascular ainda não está bem estabelecido.

Objetivo da Pesquisa:

Primário: verificar as respostas cardiovasculares agudas e crônicas ao exercício de EB em individuos com doença arterial periférica comparando-o com a atual recomendação de exercício de TC e verificar as respostas crônicas ao exercício de EB na capacidade funcional, na função cognitiva e na qualidade de vida de pacientes com doença arterial periférica.

Secundário:

a) Fase aguda: Efeitos de uma sessão aguda de TC e de EB em pacientes com doença arterial periférica sobre as respostas cardiovasculares:- índice tornozelo braço;- pressão arterial braquial;- pressão arterial ambulatorial;- pressão arterial central; - variabilidade da frequência cardíaca; - rigidez arterial;- função endotelial;

b) Fase crônica: Efeitos de um período de 12 semanas de TC e de EB sobre: • Função e

Endereço: Rua Ovídio Pires de Campos, 225 5º andar
Bairro: Cerqueira Cesar **CEP:** 05.403-010
UF: SP **Município:** SAO PAULO
Telefone: (11)2661-7585 **Fax:** (11)2661-7585 **E-mail:** cappesq.adm@hc.fm.usp.br

regulaçãocardiovascular: - índice tornozelo braço;- pressão arterial braquial;- pressão arterial central; - pressão arterial ambulatorial;- variabilidade da frequência cardíaca; - rigidez arterial, - função endotelial. • Capacidade funcional:- teste 6 minutos;- Walking Impairment Questionnaire (WIQ);- Walking Estimeted-limitation Calculed by History (WELCH);- Teste de preensão manual; Teste de marcha estacionária de dois minutos; - Short Physical Performance Battery (SPPB)- Baltimore Activity Scale for Intermittent Claudication • Cognição:- Tarefas cognitivas de função executiva e memória• Qualidade de vida:- World Health Organization Quality of Life versão curta (WHOQOL-bref);- King's College Hospital's Vascular Quality of Life Questionnaire (VASCUQOL-6).

Avaliação dos Riscos e Benefícios:

Riscos:

a) Em todos os testes que envolverem exercício físico poderá haver cansaço tanto durante quanto ao final do mesmo.

b) No teste ergométrico, em algumas pessoas que sofrem do coração, mas desconhecem este fato, esse exame poderá tornar o problema evidente. Para a segurança, este exame sempre será acompanhado de um médico; Caso haja algum problema mais grave, o paciente será encaminhado para algum especialista que poderá auxiliar no tratamento.

c) As medidas de fluxo sanguíneo podem causar um pequeno desconforto nos membros enquanto os manguitos estiverem inflados.

d) Algumas sessões poderão durar mais de 3 horas, o que pode causar um pouco de cansaço.

e) As medidas da pressão arterial ambulatorial podem chatear o paciente por ser a cada 15 minutos e durante o sono a cada 30 minutos. Isto pode também mudar o padrão do sono.

Benefícios: Os resultados do estudo podem auxiliar na utilização de exercícios alternativos para o tratamento de pacientes com doença arterial periférica. Ademais, para o participante da pesquisa o benefício deste estudo se relaciona ao fato de que o mesmo receberá gratuitamente uma avaliação cardíaca, da sua capacidade funcional, da sua cognição, a qualidade de vida e orientação específicas sobre melhores cuidados sobre a sua doença.

Comentários e Considerações sobre a Pesquisa:

Nada a acrescentar.

Considerações sobre os Termos de apresentação obrigatória:

Nada a acrescentar.

Conclusões ou Pendências e Lista de Inadequações:

Nada a acrescentar. Emenda aprovada.

Endereço: Rua Ovidio Pires de Campos, 225 5º andar
Bairro: Cerqueira Cesar **CEP:** 05.403-010
UF: SP **Município:** SAO PAULO
Telefone: (11)2661-7585 **Fax:** (11)2661-7585 **E-mail:** cappesq.adm@hc.fm.usp.br

Considerações Finais a critério do CEP:

Este parecer foi elaborado baseado nos documentos abaixo relacionados:

Tipo Documento	Arquivo	Postagem	Autor	Situação
Informações Básicas do Projeto	PB_INFORMAÇÕES_BÁSICAS_DO_PROJETO_1507979.pdf	13/02/2020 14:10:34		Aceito
Outros	Anuencia_HC_Emenda.pdf	13/02/2020 14:09:10	NELSON WOLOSKER	Aceito
Projeto Detalhado / Brochura Investigador	Projeto_limpo.docx	21/12/2019 00:18:49	NELSON WOLOSKER	Aceito
Projeto Detalhado / Brochura Investigador	Projeto_comalteracoes.docx	21/12/2019 00:18:36	NELSON WOLOSKER	Aceito
TCLE / Termos de Assentimento / Justificativa de Ausência	TCLE_cronico_limpo.docx	21/12/2019 00:18:06	NELSON WOLOSKER	Aceito
TCLE / Termos de Assentimento / Justificativa de Ausência	TCLE_cronico.docx	21/12/2019 00:17:57	NELSON WOLOSKER	Aceito
TCLE / Termos de Assentimento / Justificativa de Ausência	TCLE_agudo_limpo.docx	21/12/2019 00:17:49	NELSON WOLOSKER	Aceito
TCLE / Termos de Assentimento / Justificativa de Ausência	TCLE_agudo.docx	21/12/2019 00:17:42	NELSON WOLOSKER	Aceito
Outros	Carta_justificativa_emenda.pdf	21/12/2019 00:11:20	NELSON WOLOSKER	Aceito
Outros	Carta_justificativa_emenda.doc	21/12/2019 00:11:03	NELSON WOLOSKER	Aceito
Outros	Carta_resposta_2112.docx	21/12/2019 00:07:17	NELSON WOLOSKER	Aceito
Outros	Carta_resposta_2112.pdf	21/12/2019 00:06:00	NELSON WOLOSKER	Aceito
Outros	Carta_UNINOVE.docx	13/08/2019 15:25:35	Gabriel Grizzo Cucato	Aceito
Outros	HC.pdf	12/04/2018 12:27:59	Gabriel Grizzo Cucato	Aceito
Outros	decleb.pdf	26/02/2018	Gabriel Grizzo	Aceito

Endereço: Rua Ovídio Pires de Campos, 225 5º andar
Bairro: Cerqueira Cesar **CEP:** 05.403-010
UF: SP **Município:** SAO PAULO
Telefone: (11)2661-7585 **Fax:** (11)2661-7585 **E-mail:** cappesq.adm@hc.fm.usp.br

Outros	decleb.pdf	13:23:40	Cucato	Aceito
Outros	ResponsabilidadeInvestigadorEB.pdf	04/12/2017 13:35:13	Gabriel Grizzo Cucato	Aceito
Outros	termoparticipantesEB.pdf	04/12/2017 13:34:32	Gabriel Grizzo Cucato	Aceito
Outros	termocompromissoEB.pdf	04/12/2017 13:34:04	Gabriel Grizzo Cucato	Aceito

Situação do Parecer:
Aprovado

Necessita Apreciação da CONEP:
Não

SAO PAULO, 12 de Março de 2020

Assinado por:
ALFREDO JOSE MANSUR
(Coordenador(a))
