# Supplementary material for: Effects of arm-crank exercise on cardiovascular function, functional capacity, cognition and quality of life in patients with peripheral artery disease: Study protocol for a randomized controlled trial
Source: PLoS One. 2022 May 5;17(5):e0267849. doi: 10.1371/journal.pone.0267849 (PMC9070866; doi:10.1371/journal.pone.0267849)
Supplement: S4 File — (DOCX) [file pone.0267849.s005.docx]

RESEARCH PROJECT DATA

Research Title: Acute and chronic effects of physical exercise performed on an arm ergometer on cardiovascular function and regulation, functional, cognitive capacity and quality of life of patients with peripheral arterial disease

Researcher: NELSON WOLOSKER

Thematic Area:

Version: 1

CAAE: 81187317.6.3002.0068

Proponent Institution: Hospital das Clínicas, USP Medical School

Main Sponsor: Own Financing

OPINION DATA

Opinion Number: 3.912.388

Project presentation:

Patients with peripheral arterial disease are at high risk for fatal and non-fatal cardiovascular events. Studies with training in an arm ergometer (EB) have shown to bring benefits in the functional capacity in these patients bypassing the main barrier to the practice of exercise in these patients, pain. However, the effect of this type of training on cardiovascular function indicators is not yet well established.

Research Objective:

Primary: verify acute and chronic cardiovascular responses to EB exercise in individuals with peripheral arterial disease by comparing it with the current exercise recommendation (walking training, WT) and verify chronic responses to EB exercise in functional capacity, cognitive function and quality of life of patients with peripheral arterial disease.

Secondary:

a) Acute phase: Effects of an acute session of WT and EB in patients with peripheral arterial disease on cardiovascular responses: - ankle arm index; - brachial blood pressure; - ambulatory blood pressure; - central blood pressure; - heart rate variability; - arterial stiffness - endothelial function;

b) Chronic phase: Effects of a 12-week period of CT and EB on: • Function and cardiovascular regulation: - ankle arm index, - brachial blood pressure, - central blood pressure, - ambulatory blood pressure - heart rate variability; - arterial stiffness, - endothelial function. • Functional capacity: - 6-minute test, - Walking Impairment Questionnaire (WIQ), - Walking Estimated-limitation Calculated by History (WELCH), - Handgrip test; Two-minute stationary gait test; - Short Physical Performance Battery (SPPB) - Baltimore Activity Scale for Intermittent Claudication • Cognition: - Cognitive tasks of executive function and memory • Quality of life: - World Health Organization Quality of Life short version (WHOQOL-brief); - King's College Hospital's Vascular Quality of Life Questionnaire (VASCUQOL-6).

Assessment of Risks and Benefits:

Risks:

a) In all tests that involve physical exercise, there may be fatigue both during and at the end of it.

b) In the exercise test, in some people who suffer from the heart, but are unaware of this fact, this examination may make the problem evident. For safety, this exam will always be accompanied by a doctor; If there is a more serious problem, the patient will be referred to a specialist who can assist in the treatment.

c) Blood flow measurements can cause minor discomfort in the limbs while the cuffs are inflated.

d) Some sessions may last more than 3 hours, which can cause a little tiredness.

e) Ambulatory blood pressure measurements can upset the patient because it is every 15 minutes and during sleep every 30 minutes. This can also change the pattern of sleep.

Benefits: The results of the study can assist in the use of alternative exercises for the treatment of patients with peripheral arterial disease. Furthermore, for the research participant, the benefit of this study is related to the fact that he will receive a free cardiac evaluation, of his functional capacity, of his cognition, the quality of life and specific guidance on better care about his disease.

Research Comments and Considerations:

Nothing to add.

Considerations on the Mandatory Submission Terms:

Nothing to add.

Conclusions or Pending and List of Inadequacies:

Nothing to add. Amendment approved.
