# Supplementary material for: Effects of arm-crank exercise on cardiovascular function, functional capacity, cognition and quality of life in patients with peripheral artery disease: Study protocol for a randomized controlled trial
Source: PLoS One. 2022 May 5;17(5):e0267849. doi: 10.1371/journal.pone.0267849 (PMC9070866; doi:10.1371/journal.pone.0267849)
Supplement: S5 File — (DOCX) [file pone.0267849.s006.docx]

**Study protocol that was approved by your ethics committeeIRB**

**Título:** Efeitos do exercício físico realizado em ergômetro de braço na função e regulação cardiovascular, capacidade funcional, cognitiva e qualidade de vida de pacientes com doença arterial periférica

MATERIAL E MÉTODOS

*Desenho do estudo*

45 pacientes serão recrutados para realizarem um ensaio clínico aleatorizado e controlado com três grupos e testes pré e pós-intervenção, como demonstrado na figura 2. Esta etapa contará com cinco fases de realização: (a) recrutamento e triagem; (b) avaliação pré-intervenção; (c) adaptação aos exercícios e determinação das cargas; (d) 12 semanas de intervenção; (e) avaliação pós-intervenção.

*Questões éticas*

Este estudo já foi submetido e aprovado pelo Comitê de Ética do Hospital Israelita Albert Einstein (CAAE: 81187317.6.0000.0071.

Antes de adentrarem ao estudo, os pacientes participantes do estudo serão devidamente esclarecidos a respeito de todos os procedimentos aos quais serão submetidos, dos potenciais riscos e benefícios do estudo e aqueles que concordarem em participar assinarão o Termo de Consentimento Livre e Esclarecido.

*Amostra*

O tamanho da amostra foi determinado pelo cálculo amostral a priori específico para ensaios clínicos envolvendo grupos paralelos, neste caso ANOVA two-way com medidas repetidas, sugerido por Beck (2013) (1). Utilizando o software GPower 3.1.9.2, foi considerado um effect size de 0.25; α de 0.05; poder (1 – β) de 0.80; coeficiente de correlação entre medidas repetidas de 0.6; correção de não-esfericidade (ɛ) de 1 e número de grupos 3 e medidas 2. Desse modo, o tamanho total mínimo chegou a 36 sujeitos (12 por grupo) com poder de 83%. Considerando a possibilidade de perda amostral, a amostra foi inflada em 20%, totalizando assim uma amostra de 45 pacientes (15 por grupo). Considerando novas análises, inflaremos a amostra em 100%. Tornando assim o novo número de participantes de 90 (30 por cada grupo).

*Riscos e benefícios do estudo*

Os riscos dos estudos agudo e crônico podem estar relacionados a alguns aspectos. 1) Em todos os testes que envolverem exercício físico poderá haver cansaço tanto durante quanto ao final do mesmo. 2) A medida em que o manguito ficará apertando no braço por 5 minutos pode gerar incômodo ou dor no paciente. O benefício deste estudo se relaciona ao fato de que o paciente irá receber gratuitamente uma avaliação do seu coração e orientações específicas sobre melhores cuidados sobre a sua doença.

## Recrutamento e Triagem

Serão convidados a participar pacientes de ambos os gêneros com DAP recrutados nos Hospitais de São Paulo. Após aceitar participar do estudo, cada paciente será submetido a uma avaliação clínica. Os pacientes que atenderem aos critérios de inclusão participarão, então, das etapas posteriores.

Os pacientes responderão a um questionário para obtenção de informações referentes à idade, sexo e fatores de risco cardiovascular (presença de doenças como diabetes, hipertensão, dislipidemia, tabagismo e nível de atividade física). Para informações sobre as medicações em uso, será solicitado que todos os pacientes tragam para o laboratório todos os medicamentos e as receitas a fim de que se possa ter o controle da classe medicamentosa, dosagem e a frequência de uso. Serão também obtidas a massa corporal, estatura e circunferências corporais.

Para confirmação do diagnóstico da DAP será realizada a medida do índice tornozelo braço por meio das pressões arteriais sistólicas do braço e do tornozelo nos dois membros medidas em triplicata. A aferição da pressão arterial será realizada com o uso de um doppler vascular (DV610, Medmega, Brasil) e um esfigmomanômetro aneroide devidamente calibrado. Após posicionar o doppler sobre a artéria braquial (para a medida no braço) e sobre as artérias pediosa e tibial posterior (no tornozelo), o manguito será inflado até 20 mmHg acima do nível estimado da pressão arterial sistólica. A determinação da pressão arterial sistólica será feita no momento do aparecimento do primeiro som (fase I de Korotkoff). As medidas no braço e tornozelo serão realizadas por um único avaliador. Em posse desses dados, será calculado o índice tornozelo braço de cada lado do corpo por meio da divisão da pressão arterial sistólica do tornozelo (maior pressão arterial sistólica entre os dois pontos anatômicos medidos) pela pressão arterial sistólica do braço (maior pressão arterial sistólica).

Para identificar se os indivíduos estão aptos a realização do exercício físico, os pacientes serão submetidos a um teste ergométrico máximo com protocolo específico para essa população (2). O teste será iniciado com velocidade constante de 3,2 km/h, com incrementos de 2% de inclinação a cada dois minutos até a exaustão. Durante o teste, a frequência cardíaca será continuamente monitorada por um eletrocardiograma. Caso sejam identificadas respostas anormais ou sugestivas de doenças isquêmicas cardíacas ao exercício, o paciente não participará do protocolo do estudo.

Serão incluídos no estudo o paciente que: apresente DAP, confirmado pelo diagnóstico do índice tornozelo braço <0,90 em um ou em ambos os membros; apresente idade ≥ 40 anos, se mulher estar na pós-menopausa sem uso de terapia de reposição hormonal; não seja tabagista ativo, e; estejam aptos à prática de exercício físico.

Os pacientes aptos a participarem do estudo só serão excluídos caso façam alguma mudança de medicação antes de completar o período de intervenção ou apresentem algum comprometimento na saúde que contraindique a prática de exercício físico.

*Aleatorização dos grupos, sigilo da alocação e cegamento*

Todos os pacientes serão alocados aleatoriamente para os sub-grupos experimentais: treinamento em EB, treinamento de caminhada (TC) e controle (GC). Para tanto, será realizada a aleatorização por blocos de cada nove pacientes, que serão ordenados de acordo com seus níveis de pressão arterial clínica. A partir disso, os pacientes serão sorteados três a três para comporem os três grupos experimentais. Os números para a aleatorização serão gerados no site *www.randomizer.org* de modo que conste a mesma quantidade de pacientes nos três grupos. O pesquisador responsável pela alocação não será responsável pelo recrutamento e fará a aleatorização apenas quando nove pacientes tiverem sido incluídos consecutivamente, garantindo assim o sigilo na alocação. Vale ainda destacar que o pesquisador que realizará as análises será cego para os grupos que os participantes serão designados.

*Adaptação e Determinação das cargas*

Os pacientes randomizados para o sub-grupo EB participarão de uma sessão de adaptação ao aparelho de EB. Para essa familiarização, os pacientes realizarão 10 séries com menor intensidade possível do equipamento, com dois minutos de duração e dois minutos de intervalo passivo entre elas. Para a determinação das cargas o paciente realizará um teste progressivo no aparelho de EB, mantendo a cadência de 50 rotações por minuto durante todo o teste. Inicialmente o paciente terá um aquecimento de dois minutos de duração com a carga mínima oferecida pelo aparelho. A partir disso, haverá um acréscimo de 10 watts a cada minuto do teste. O paciente tentará manter a frequência de 50 rotaçõs por minuto a cada ciclo do exercício com dois minutos de duração e dois minutos de intervalo. Quando for encontrada a carga em que as intensidades sejam equivalentes a 13 – 15, considerada uma intensidade cansativa, na escala de percepção subjetiva de esforço (Escala de Borg – 6 à 20), o teste será finalizado (3, 4). A frequência cardíaca será monitorada continuamente por um eletrocardiograma e a percepção subjetiva de esforço será obtida ao fim de cada minuto do exercício.

Os pacientes randomizados para o sub-grupo TC participarão de uma sessão de adaptação à esteira ergométrica. Para essa familiarização, os pacientes realizarão 10 séries à 3,2km/h e sem nenhuma inclinação, com dois minutos de duração e dois minutos de intervalo entre elas. Para a determinação das cargas o paciente realizará um teste progressivo na esteira. Inicialmente o paciente terá um aquecimento de dois minutos à 3,2km/h e sem inclinação. A partir disso, haverá um acréscimo de 0,3km/h a cada minuto do teste. Cada ciclo do exercício contará com dois minutos de duração e dois minutos de intervalo passivo. Quando for encontrada velocidade em que as intensidades sejam equivalentes a 13 – 15, considerada uma intensidade cansativa, na escala de percepção subjetiva de esforço (Escala de Borg – 6 à 20), o teste será finalizado (3, 4). A frequência cardíaca será monitorada continuamente por um eletrocardiograma e a percepção subjetiva de esforço será obtida ao fim de cada minuto do exercício.

*Treinamento em Ergômetro de Braço e Treinamento de Caminhada*

Ambos treinamentos em EB e caminhada serão composto por duas sessões semanais de exercício durante 12 semanas. O exercício será realizado de forma intervalada. Nas primeiras semanas de treinamento, cada sessão será composta por 15 ciclos, sendo dois minutos ativos e dois minutos de intervalo passivo, totalizando 60 minutos por sessão (30 minutos ativos). Após as três primeiras semanas de treinamento, o tempo do exercício será acrescido, progressivamente, em um minuto a cada 3 semanas e o período de recuperação será decrescido, completando ao final um volume máximo de 10 séries, cinco minutos de exercício e um minuto de recuperação passaiva por 59 minutos, sendo 50 minutos ativos. A progressão dos tempos ativos, tempos de intervalo passivo e volume total do treino, de acordo com cada sermana, está demonstrado no quadro 1. A intensidade do exercício será determinada pela carga equivalente ao intervalo de 13 – 15, considerada uma intensidade cansativa, na escala de percepção subjetiva de esforço (Escala de Borg – 6 à 20) (4). A avaliação da intensidade será feita durante (a cada série de exercício) e ao final de cada sessão de treinamento. Deve-se ressaltar que a prescrição desse protocolo de treinamento já se mostrou eficaz em melhorar a capacidade funcional após 12 semanas de treinamento em EB e TC em pacientes com DAP (3).

| **Semanas** | **Número de Ciclos** | **Tempo Ativo** | **Tempo Total Ativo** | **Tempo de Intervalo** | **Tempo Total de Intervalo** | **Tempo Total da Sessão** |
| --- | --- | --- | --- | --- | --- | --- |
| 1-3 Semana | 15 | 2min | 30 min | 2 min | 30 min | 60 min |
| 4-6 Semana | 14 | 3min | 42 min | 1 min 30 s | 19 min 30 s | 62 min |
| 7-9 Semana | 12 | 4 min | 48 min | 1 min | 11 min | 59 min |
| 10-12 Semana | 10 | 5min | 50 min | 1 min | 9 min | 59 min |

**Quadro 1.** Periodização do treinamento no período das 12 semanas.

Os pacientes aleatorizados para o sub-grupo GC participarão de reuniões com os pesquisadores também duas vezes na semana, durante as 12 semanas. Nessas reuniões os pacientes realizarão tarefas manuais, com uso ou não de materiais artísticos, programações culturais, aulas de culinária e cuidado com a casa, entretanto sem nenhum componente de exercício. Essa prática do GC será realizada com o objetivo de minimizar os efeitos do compromisso e deslocamento bissemanal do paciente até o local de treino, minimizar a influência do contato paciente-pesquisador e minimizar o efeito convívio entre os próprios pacientes, o que haverá nos grupo EB e TC.

Todos os pacientes que forem incluídos no estudo, em todos os grupos, também receberão recomendações para aumentar os níveis de atividade física, uma vez que essa é a recomendação padrão do tratamento clínico para esses pacientes (5).

*Avaliações pré e pós-intervenção*

- *Avaliações cardioavasculares*

As avaliações cardiovasculares serão realizadas no período pré-intervenção e no período pós-intervenção. Os dados serão obtidos por um pesquisador devidamente treinado e experiente. Antes das coletas os pacientes receberão orientações para manter o padrão de sono, não realizar exercício físico ou consumir bebidas que contenham cafeína e alcoólicas 24 horas antes das avaliações.

*Índice tornozelo braço*

O índice tornozelo braço será calculado para cada lado do corpo por meio da divisão da pressão arterial sistólica do tornozelo (maior pressão arterial sistólica medida entre os dois pontos anatômicos) pela maior pressão arterial sistólica encontrada entre os braços. A aferição da pressão arterial braquial será avaliada por meio de um monitor (HEM-742, Omron Healthcare, Japão), a do tornozelo será realizada com o uso de um doppler vascular (DV610, Medmega, Brasil) e um esfigmomanômetro aneroide devidamente calibrado. Após posicionar o doppler sobre as artérias pediosa e tibial posterior (no tornozelo), o manguito será inflado até 20 mmHg acima do nível estimado da pressão arterial sistólica. A determinação da pressão arterial sistólica será feita no momento do aparecimento do primeiro som (fase I de Korotkoff). As medidas no braço e tornozelo serão realizadas por um único avaliador. Em posse desses dados, será calculado o índice tornozelo braço de cada lado do corpo por meio da divisão da pressão arterial sistólica do tornozelo (maior pressão arterial sistólica entre os dois pontos anatômicos medidos) pela pressão arterial sistólica do braço (maior pressão arterial sistólica).

*Pressão arterial braquial*

A pressão arterial braquial será avaliada por meio de um monitor (HEM-742, Omron Healthcare, Japão) o qual consiste em um aparelho eletrônico e digital da medida da pressão arterial de braço, com deflação e inflação automática de ar. Para essa medida, os indivíduos permanecerão por dez minutos na posição supina. Serão realizadas três medidas consecutivas, com um minuto de intervalo, em ambos os braços e com tamanho do manguito adequado para a circunferência do braço. O valor utilizado será a média das duas últimas medidas, conforme recomendado pela Sociedade Brasileira de Cardiologia (6).

*Pressão arterial central*

A medida da pressão arterial central será realizada pela análise da onda de pulso da artéria radial por meio da técnica de tonometria de aplanação (SphygmoCor, AtCor Medical, Australia). Para tanto, após a medida da pressão arterial braquial, também na posição supina, serão utilizados 11 segundos de gravação da onda da pressão arterial radial. Após esse procedimento o Software SphygmoCor® derivará a onda de pressão da aorta ascendente, equivalente à onda de pressão medida por um cateter invasivo obtendo-se os valores de pressão arterial central sistólica, diastólica e média (6).

*Monitorização ambulatorial da pressão arterial*

A medida ambulatorial da pressão arterial será realizada no braço não dominante do paciente por um monitor automático, modelo Dynamapa (Cardio Sistemas Comercial e Industrial Ltda, Brasil), que será programado para realizar medidas a cada 15 minutos no período de vigília e a cada 30 minutos no período de sono. Serão considerados válidos apenas os registros que tenham pelo menos 80% das medidas válidas. Essa análise segue a recomendação das IV Diretriz Brasileira para Uso da Monitorização Ambulatorial da Pressão Arterial.(2)

*Variabilidade da frequência cardíaca*

A variabilidade da frequência cardíaca será avaliada a fim de estimar a modulação autonômica do sistema cardiovascular. Para tanto, após 20 minutos de repouso, os pacientes permanecerão 10 minutos na posição supina, período no qual terão registrados os intervalos RR, por meio de um monitor de frequência cardíaca válido para esta função (V800, Polar Electro, Finlândia). Será considerado um sinal válido aqueles com pelos menos cinco minutos de sinal estacionário.

Após a coleta, os intervalos RR serão exportados para o programa Kubios HRV (Version 2.0, Biosignal Analysis and Medical Imaging Group, Finlândia), cujas análises serão realizadas (domínio do tempo e da frequência). Os parâmetros do domínio do tempo, desvio padrão de todos os intervalos RR (SDNN), raiz quadrada da média do quadrado das diferenças entre os intervalos RR normais adjacentes (RMSSD) e porcentagem dos intervalos adjacentes com mais de 50ms (PNN50) serão obtidos. Os parâmetros do domínio da frequência serão obtidos pela técnica da análise espectral, utilizando o método autorregressivo, com a ordem do modelo de 12 pelo critério de Akaike. Serão consideradas como fisiologicamente significativas as frequências entre 0,04 e 0,4 Hz, sendo o componente de baixa frequência (BF) representado pelas oscilações entre 0,04 e 0,15 Hz e o componente de alta frequência (AF) entre 0,15 e 0,4 Hz(7).

*Rigidez arterial*

A estimativa da rigidez arterial central será obtida a partir da avaliação da velocidade da onda de pulso seguindo as orientações da Clinical Application of Arterial Stiffness, Task Force III (8). Para tanto, será utilizada a técnica de tonometria de aplanação. As ondas de pulso carótido-femoral serão registradas sequencialmente por transdutores transcutâneos posicionados acima das artérias utilizando-se um aparelho de tonometria por aplanação (SphygmoCor, AtCor Medical, Australia). O registro eletrocardiográfico será obtido simultaneamente às medidas como padrão de referência para calcular o tempo de trânsito da onda de acordo com o método “foot-to-foot”. As distâncias entre superfícies serão medidas pelo investigador: entre o ponto de gravação da artéria carótida e da fúrcula esternal, entre a fúrcula esternal e o ponto de gravação na artéria femoral. A distância percorrida pela onda de pulso será calculada pela subtração distância mais distal e a mais proximal. A velocidade da onda de pulso será calculada como = distância percorrida pela onda de pulso (m) / tempo de trânsito (s)

*Fluxo sanguíneo e função endotelial*

As medidas de fluxo sanguíneo basal e vasodilatação mediada pelo fluxo serão obtidas por meio de ultrassonografia de acordo com as recomendações mais recentes. Serão registradas imagens da artéria braquial e femoral por um aparelho de ultrassonografia bidimensional com Doppler espectral e transdutor linear (Ultra-0122, Philips, Holanda).

A resolução de contraste, profundidade, e ganho serão ajustadas para otimizar as imagens longitudinais da interface parede lúmen/arterial. Os espectros de velocidade do fluxo sanguíneo serão registrados simultaneamente através do modo pulsátil em frequência linear de 10 MHz com o ângulo de inclinação de 60º.

O diâmetro e o fluxo sanguíneo basal serão registrados continuamente ao longo de 90 segundos. Após a gravação basal, será inflado um manguito, posicionado no antebraço (avaliação da artéria braquial) ou logo acima do joelho (avaliação da artéria femoral), o qual estará com uma pressão 50 mm Hg acima da pressão arterial sistólica, medida previamente ao exame. A oclusão será mantida por 5 minutos e, após esse período, liberada rapidamente. As gravações do doppler serão retomadas 30 segundos antes de desinflar o manguito e serão mantidas por mais 180 segundos.

O diâmetro e o fluxo sanguíneo pós-oclusão será medido após a liberação. A capacidade vasodilatadora será calculada como o percentual de aumento do diâmetro da artéria pós-oclusão em relação aos seus valores basais.

Todas as imagens capturadas serão transferidas ao computador por meio de uma placa de captura de vídeo USB (Easy Cap, Leadership, Brasil) na frequência de 30 Hz e salvas em disco rígido externo para posterior análise através de um software semiautomático (FMD Studio, Institute of Clinical Physiology, Itália). O diâmetro basal será definido como a média de 60 segundos de dados obtidos no momento anterior a insuflação do manguito. O diâmetro pico será definido como o maior valor de diâmetro encontrado após a liberação do manguito. A vasodilatação mediada pelo fluxo da artéria braquial será calculada como a diferença percentual entre o diâmetro basal e o diâmetro pico. Os dados serão apresentados pelo software como taxa de cisalhamento anterógrado (SR_ANT_) e taxa de cisalhamento retrógrado (SR_RET_). A taxa de cisalhamento média (SR_MEAN_) será calculada como a diferença entre os componentes anterógrado e retrógrado. A área sob a curva da taxa de cisalhamento (AUC_SR_), indicativo do estímulo para vasodilatação, será calculada do momento da liberação do manguito até o diâmetro de pico ser atingido. O fluxo sanguíneo será calculado a partir da fórmula “Fluxo=Vmπ(D^2^/4)x60”, na qual, Vm corresponde a velocidade média do fluxo sanguíneo em centímetros por segundo e D representa o diâmetro da artéria braquial em centímetros (9). A velocidade média será obtida pela fórmula “Vm=(SR_MEAN_ x D)/4”. O índice de oscilação do fluxo será determinado como “SR_RET_/(SR_ANT_+SR_RET_)”, sendo que os valores obtidos variam de 0 a 0,5 indicando fluxo laminar unidirecional e pura oscilação, respectivamente (10).

- *Avaliações da capacidade funcional*

*Testes de 6 minutos*

Para a avaliação da capacidade funcional será utilizado teste de 6 minutos em um corredor de 30 metros, seguindo o protocolo previamente descrito (11). Para a realização do teste, os pacientes serão instruídos a completar tantas voltas quanto forem possíveis. Será permitido ao paciente interromper a caminhada durante o teste caso o sintoma de CI se tornar intolerável, porém o cronômetro não será interrompido durante este momento. Os pacientes que pararem de caminhar por conta dos sintomas de CI serão encorajados a retornar à caminhada tão breve quanto possível. Ao final do teste, quando for atingido o tempo de seis minutos, será identificado a distância total de caminhada.

*Walking Impairment Questionnaire (WIQ)*

O WIQ é um questionário que fornece indicadores sobre a capacidade de caminhada, de forma subjetiva, de pacientes com CI em situações cotidianas, sendo composto por quatro domínios: diagnóstico diferencial, distância de caminhada, velocidade de caminhada e capacidade de subir escadas (12).

Para o diagnóstico diferencial, perguntas referentes ao grau de dores e outros sintomas são analisadas, sendo que 0 representa nenhuma dificuldade/e ou ausência do sintoma e 4 muita ou extrema dificuldade. Para a distância de caminhada, a cada distância, o qual varia de 5 metros a 450 metros, é analisado o grau de dificuldade do paciente, sendo que 0 representa nenhuma dificuldade e 4 incapaz. Com relação a velocidade de caminhada, diferentes graus de velocidade, que variam de 3,2 km/h a 8 km/h são analisados em termos de dificuldade, sendo que 0 representa nenhuma dificuldade em caminhar em certa distância e 4 incapaz. Por fim, a dificuldade de subir escadas é analisada pela quantidade de degraus, que varia de 8 degraus a 24 degraus, com sua dificuldade de realização desta tarefa, sendo que 0 representa nenhuma dificuldade e 4 incapaz.

Para análise da pontuação do WIQ, para cada pergunta é analisado o grau de dificuldade e o seu "peso", o qual é multiplicado. Os produtos são somados e divididos pela máxima pontuação possível para obter a pontuação total do WIQ.

*Walking Estimated-Limitation Calculated by History (WELCH)*

O questionário WELCH apresenta quatro perguntas que estão relacionadas com a velocidade e o tempo, subjetivos, em que o paciente consegue caminhar em comparação aos seus parentes, amigos ou indivíduos da mesma idade. Para cada pergunta, o paciente deverá assinalar apenas uma resposta. Caso haja mais de uma resposta, o avaliador irá novamente explicar o questionário, e o paciente deverá preenchê-lo novamente.

Para o cálculo da pontuação do WELCH, será considerado para as 3 primeiras perguntas: (0) para impossível; (1) para 30 segundos; (2) para 1 minuto; (3) para 3 minutos; (4) para 10 minutos; (5) para 30 minutos; (6) para 1 hora e (7) para mais de 3 horas. Para a quarta pergunta, será considerado como coeficiente: (1) para muito devagar; (2) para moderadamente devagar; (3) um pouco devagar, (4) na mesma velocidade; (5) mais rápido. A pontuação final será definida como:

Valor total= [(valor resposta 1 + valor resposta 2 + valor resposta 3) -1] X coeficiente da pergunta 4.

*Teste de Preensão Manual*

O teste de força de preensão manual, é realizado através de um dinamômetro de preensão manual que representa a força global de indivíduos idosos . Os pacientes serão avaliados sentados com o ombro levemente aduzido, o cotovelo fletido a 90°, o antebraço e o punho em posição neutra, conforme preconiza a *American Society of Hand Therapists* (13). A contração voluntária máxima de preensão manual será obtida utilizando um dinamômetro com display digital (EH101, CAMRY, China) ajustável e calibrado com escala de 0 a 100 kgf.

Os indivíduos receberão as orientações necessárias previamente ao teste realizando algumas contrações, até que se sentir familiarizado com o equipamento. Será realizado o ajuste do posicionamento das mãos, de modo que a articulação interalangeana proximal da mão seja ajustada sob a barra para que permita que a preensão seja realizada entre os dedos e a região tênar com máximo conforto. Será orientado que que o braço permaneça imóvel, havendo apenas flexão das articulações da mão.

O teste da contratação voluntária máxima será realizado em três tentativas para ambos os braços. Em cada tentativa os indivíduos realizarão força durante cinco segundos com intervalo de recuperação entre cada tentativa de um minuto. Incentivos verbais serão dados para os indivíduos realizarem força no teste.

*Teste de marcha estacionária de dois minutos*

O teste de marcha estacionária consiste em um teste em que o indivíduo fica em pé, com os pés juntos e eleva os joelhos até uma altura em que a perna e a coxa façam um ângulo de aproximadamente 90 graus. Esse movimento é repetido por dois minutos e é avaliada a quantidade de vezes que o paciente consegue realizar tais repetições.

Este teste será realizado em local plano e, para tanto, será identificada a altura mínima que o joelho deveria alcançar individualmente, padronizada a partir do ponto médio entre a patela e a espinha ilíaca antero-superior. Nesta altura será colocado um elástico, para que o paciente possa ter uma referência de onde deve alcançar com o joelho.

Para a realização do teste os pacientes serão instruídos a completar tantos passos quanto forem possíveis durante o período de 2 minutos. A cada 30 segundos será dado um comando verbal para encorajar o desempenho: “o senhor está indo muito bem continue assim”. Será permitido ao paciente interromper a marcha durante o teste caso o sintoma de CI se tornar intolerável, porém o cronômetro não será interrompido durante este momento. Os pacientes que pararem de marchar por conta dos sintomas de CI serão encorajados a retornar à marcha tão breve quanto possível. O avaliador quantificará o número de elevações.

*Short Physical Performance Battery (SPPB)*

A capacidade funcional também será obtida pela Short Physical Performance Battery (14), composto por quatro testes que avaliam o equilíbrio, a marcha e a força de membros inferiores.

O equilíbrio é avaliado em três posições dos pés: 1. em paralelo, 2. com o hálux encostado na borda medial do calcanhar e 3. com o hálux encostado na borda posterior do calcanhar. Atribui-se 1 ponto se realizado em tempo <10" e zero se for >10", para os dois primeiros testes. No terceiro teste, a pontuação varia de zero para <3", 1 entre 3" e 9,99" e 2 se for >10".

Para a avaliação da marcha, é utilizado um cronômetro para registrar o tempo que o indivíduo levou para percorrer um corredor de 4 metros (ida e volta), repetindo duas vezes o percurso. A pontuação do instrumento varia de: zero, quando incapaz; 1, se >8,70"; 2 se o tempo varia entre 6,21" e 8,70"; 3 entre 4,82" e 6,20"e 4, se tempo <4,82".

A força muscular dos membros inferiores é obtida por meio do tempo que o idoso levou para levantar-se de uma cadeira com os membros superiores cruzados sobre o peito, repetindo o teste cinco vezes consecutivas. Os escores variam de acordo com tempo gasto: zero quando incapaz; 1, se > 16,7"; 2 entre 13,7" e 16,69"; 3, tempo entre 11,2" e 13,69" e 4 se tempo <11,19".

O escore total da bateria de teste, obtido pela soma das pontuações de cada teste permite valores entre zero a 12 pontos.

*Baltimore Activity Scale for Intermittent Claudication*

O Baltimore Activity Scale for Intermittent Claudication é composto por 5 questões, referentes aos sintomas de CI. Para cada pergunta, o paciente seleciona a resposta que melhor descreve seus sintomas e o nível de atividade física. Os valores variam de 0 a 2 pontos, e a pontuação total é a soma dos pontos das 5 perguntas. O escore varia de 0 a 10, sendo que zero seria os menores níveis de atividade física e dez o maior.

- *Qualidade de Vida*

*World Health Organization Quality of Life (WHOQOL)bref*

O instrumento *WHOQOL-bref* engloba diferentes aspectos da saúde física e mental, que englobam as dimensões: estado geral de saúde, capacidade funcional, aspectos físicos, dor, vitalidade, saúde mental, aspectos emocionais e aspectos sociais da vida. Cada uma das respostas recebe uma pontuação, que são somadas a uma constante para determinar os diferentes componentes da qualidade de vida.

O estilo de vida será avaliado por meio de uma versão adaptada do questionário Pentáculo do bem-estar, que engloba questões referentes aos componentes: nutrição, atividade física, comportamento preventivo, relacionamento social e controle do estresse. Para cada pergunta, os pacientes responderão se tal comportamento não faz parte do seu estilo de vida, raramente corresponde, quase sempre corresponde ou sempre corresponde.

*King’s College Hospital’s Vascular Quality of Life Questionnaire (VASCUQOL-6)*

*O King’s College Hospital’s Vascular Quality of Life* é um questionário específico para avaliação da qualidade de vida em pacientes com DAP. Esse questionário é composto de 6 questões que englobam as limitações físicas e socais decorrentes dos sintomas de dor dos membros inferiores desses pacientes. Cada questão apresenta 4 opções de respostas. A pontuação do questionário é calculada somando os resultados de cada questão, sendo que os valores podem variar de 6 a 24 pontos.

- *Capacidade Cognitiva*

O paciente passará por algumas tarefas cognitivas padronizadas para quantificar a função executiva e a memória. Essas avaliações serão realizadas em um papel e incluem: a) função executiva – Teste A e B, codificação de símbolos de dígitos; b) memória: teste de aprendizado verbal de Hopkins (recuperação imediata e atrasada), intervalo de dígitos para frente e para trás; c) fluência verbal: tarefa de geração de palavras ("S" e animais).

*Controle das variáveis intervenientes*

Com o objetivo de minimizar as possíveis influências de variáveis intervenientes e poder assegurar que as modificações geradas no sistema cardiovascular foram causadas pelo período de treinamento em EB, haverá o controle das medicações, acompanhamento do padrão alimentar e do nível de atividade física dos pacientes.

Para controle das medicações, a cada semana o pesquisador ficará responsável de preencher um recipiente com os medicamentos prescritos para o paciente, de acordo com a frequência, o horário e dosagem recomendada. Ao final da semana, o pesquisador conferirá se o paciente usou corretamente suas medicações durante a semana que se passou.

Para o acompanhamento alimentar, antes e na última semana do programa de treinamento será pedido que o paciente faça um recordatório alimentar durante quatro dias da semana, incluindo pelo menos um dia do final de semana. Com base nas informações obtidas pelos pacientes, será possível analisar possíveis mudanças no padrão alimentar dos pacientes, bem como estimar o consumo calórico ingerido antes e após o período de treinamento.

Por fim, os pacientes utilizarão um relógio que monitorará os níveis de atividade física POLAR A300 (Polar, Filândia). O POLAR A300 trata-se de um monitor acoplado com um acelerômetro 3D que registra os movimentos dos pacientes. Por ele é possível analisar a frequência, intensidade e regularidade dos movimentos e determinar o nível de atividade física. Os pacientes usarão esse relógio monitor por uma semana tanto antes da primeira avaliação quanto na última semana de treinamento, permitindo-nos identificar possíveis alterações no padrão de nível de atividade física com as intervenção nos grupos EB e GC.

*Análise estatística*

As análises de normalidade e homogeneidade de variância serão realizadas por meio dos testes de Shapiro-Wilks e Levene, respectivamente. Para comparação das variáveis no momento pré-intervenção será utilizada a ANOVA one way.

Antes e após o período de intervenção, será utilizada análise de variância de dois caminhos para medidas repetidas, tendo como fatores principais o sub-grupo (EB, TC e GC) e o tempo (pré e e pós-intervenção). Em todas as análises, quando for verificado efeito significante será empregado o teste de post-hoc de Newman-Keuls. Será considerado significante o valor de *P*<0,05 e os dados serão apresentados em média ± desvio padrão.

# **REFERÊNCIAS**

1. Beck TW. The importance of a priori sample size estimation in strength and conditioning research. Journal of strength and conditioning research / National Strength & Conditioning Association. 2013;27(8):2323-37.

2. Gardner AW, Skinner JS, Cantwell BW, Smith LK. Progressive vs single-stage treadmill tests for evaluation of claudication. Medicine and science in sports and exercise. 1991;23(4):402-8.

3. Treat-Jacobson D, Bronas UG, Leon AS. Efficacy of arm-ergometry versus treadmill exercise training to improve walking distance in patients with claudication. Vasc Med. 2009;14(3):203-13.

4. Borg GA. Psychophysical bases of perceived exertion. Medicine and science in sports and exercise. 1982;14(5):377-81.

5. Brook RD, Appel LJ, Rubenfire M, Ogedegbe G, Bisognano JD, Elliott WJ, et al. Beyond medications and diet: alternative approaches to lowering blood pressure: a scientific statement from the american heart association. Hypertension. 2013;61(6):1360-83.

6. Inder JD, Carlson DJ, Dieberg G, McFarlane JR, Hess NC, Smart NA. Isometric exercise training for blood pressure management: a systematic review and meta-analysis to optimize benefit. Hypertension research : official journal of the Japanese Society of Hypertension. 2015.

7. Pini R, Cavallini MC, Palmieri V, Marchionni N, Di Bari M, Devereux RB, et al. Central but not brachial blood pressure predicts cardiovascular events in an unselected geriatric population: the ICARe Dicomano Study. Journal of the American College of Cardiology. 2008;51(25):2432-9.

8. Van Bortel LM, Duprez D, Starmans-Kool MJ, Safar ME, Giannattasio C, Cockcroft J, et al. Clinical applications of arterial stiffness, Task Force III: recommendations for user procedures. American journal of hypertension. 2002;15(5):445-52.

9. Grizzo Cucato G, de Moraes Forjaz CL, Kanegusuku H, da Rocha Chehuen M, Riani Costa LA, Wolosker N, et al. Effects of walking and strength training on resting and exercise cardiovascular responses in patients with intermittent claudication. VASA Zeitschrift fur Gefasskrankheiten. 2011;40(5):390-7.

10. Gomes APF, Prazeres TMPd, Correia MdA, Santana FdS, Farah BQ, Ritti-Dias RM. Cardiovascular responses of peripheral artery disease patients during resistance exercise. Jornal Vascular Brasileiro. 2015;14:55-61.

11. Montgomery PS, Gardner AW. The clinical utility of a six-minute walk test in peripheral arterial occlusive disease patients. J Am Geriatr Soc. 1998;46(6):706-11.

12. Ritti-Dias RM, Gobbo LA, Cucato GG, Wolosker N, Jacob Filho W, Santarem JM, et al. Translation and validation of the walking impairment questionnaire in Brazilian subjects with intermittent claudication. Arquivos brasileiros de cardiologia. 2009;92(2):136-49.

13. Silva GO, Farah BQ, Germano-Soares AH, Andrade-Lima A, Santana FS, Rodrigues SL, et al. Acute blood pressure responses after different isometric handgrip protocols in hypertensive patients. Clinics. 2018;73: e373.

14. Guralnik JM, Simonsick EM, Ferrucci L, Glynn RJ, Berkman LF, Blazer DG, et al. A short physical performance battery assessing lower extremity function: association with self-reported disability and prediction of mortality and nursing home admission. J Gerontol. 1994;49(2): M85-94.
