# Supplementary material for: Effects of arm-crank exercise on cardiovascular function, functional capacity, cognition and quality of life in patients with peripheral artery disease: Study protocol for a randomized controlled trial
Source: PLoS One. 2022 May 5;17(5):e0267849. doi: 10.1371/journal.pone.0267849 (PMC9070866; doi:10.1371/journal.pone.0267849)
Supplement: S6 File — (DOCX) [file pone.0267849.s007.docx]

**Translation of the study protocol that was approved by your ethics committeeIRB**

**Effects of arm-crank exercise on functional capacity, cardiovascular function, cognition and quality of life in patients with peripheral artery disease: Study protocol for a randomized controlled trial**

**Materials and Methods**

**Eligible participants**

Patients with symptomatic PAD will be recruited from Hospitals of São Paulo, Brazil. Patients will be included if they present a) Age > 40 years old; b) Ankle brachial index (ABI) < 0.90 in one or both limbs; c) if woman in post-menopausal period without hormone replacement therapy; and d) able to perform physical exercise in upper and lower limbs.

After agreeing to participate in the study, each patient will undergo a clinical evaluation. To confirm PAD diagnosis, ABI will be assessed in accordance with previous guidelines [1]. Briefly, ABI will be measured as the highest systolic blood pressure in the posterior tibial or dorsalispedis artery divided by the highest systolic blood pressure in the brachial artery. Blood pressure measurements will be recorded in both limbs using a doppler vascular monitor (DV160, Medmega, Brazil) and a sphygmomanometer.

To identify possible cardiovascular abnormalities during exercise, patients will be submitted to maximal treadmill test with a specific protocol for PAD population [2]. The test will start at a constant speed of 3.2 km/h, with 2% incline increments every two minutes until exhaustion. During the test, heart rate will be continuously monitored by an electrocardiogram. BP will be obtained at every two minutes using a mercury sphygmomanometer. It will be included only patients with no restrictions to perform physical exercise training.

**Study design**

This is randomized clinical trial with single-blind data collection. The protocol study will be conducted according to the principles of the Declaration of Helsinki and with the ethical principles governing research involving human subjects stipulated in Resolution 466/2012 of the Brazilian National Board of Health. The study protocol was approved by the Research Ethics Committee of Human Research of Hospital Israelita Albert Einstein, Brazil (February 2020 - CAAE: 81187317.6.0000.0071). All participants will read and sign an informed consent form before enrollment. Participation will be voluntary, and all ethical principles of confidentiality and data protection will be maintained.

Patients will perform two visits with an interval of at least 48 hours. In the first visit, patients will perform the functional tests (i.e. 6-min walk test, 2 minute step test [2 MST], handgrip test, walking impairment questionnaire [WIQ], walking estimated limitation calculated by history [WELCH], Baltimore activity scale for intermittent claudication, and short physical performance battery [SPPB]), cognitive assessment and answer the quality of life questionnaires (vascular quality of life questionnaire [VASCUQOL-6] and World Health Organization Quality of Life [WHOQOL brief]). On the second visit, patients will undergo cardiovascular assessments (i.e. office BP, ambulatory BP, central BP, heart rate variability, arterial stiffness and vascular function). After that, patients will be randomly placed in blocks to one of the three intervention groups (WT, ACT and control groups [CG]) and will be re-evaluated after 12 weeks. The random allocation sequence will be generated by Researcher Randomizer (https://www.randomizer.org). These data will be collected by a trained kinesiologist who will be blind to interventions.

During the study, patients will be excluded if they change their medication or present any health impairment that contraindicates the continuation of the practice of physical exercise during the study.

**Interventions – Arm Crank Exercise, Treadmill Exercise, and Control Group**

Before starting the program, patients randomized to the ACT group will perform an adaptation session to familiarize with the ergometer and also to identify the training load. For this, patients will perform 10 bouts of two minutes exercising with minimum load with two minutes of passive interval between them. Patient will be instructed to maintain 50 revolutions per minute (RPM) during exercise. After that, to determine the training load for each session, patient will perform a progressive test where the load will be increased in 10 watts every minute of the test, following the protocol of two minutes of exercise with two minutes of interval until the patient report 13 – 15 values on the subjective effort perception scale (Borg scale - 6 to 20) [3].

Similarly, patients randomized to the WT group will perform 10 bouts of two minutes at 3.2 km/h without grade in the treadmill. After that, the load will be progressively increased in 0.3 km/h every minute of the test until the patient report 13 – 15 values at Borg scale. In both adaptation (ACT and WT), heart rate will be continuously monitored via heart rate monitor (Polar A300, Polar, Finland) and the subjective effort perception scale (Borg scale - 6 to 20) will be obtained at the end of each minute during the exercise.

Thus, during the first training sessions (i.e. ACT and WT), the workload corresponding to 13-15 values at Borg scale obtained in the progressive test will be employed. Arm Crank exercise Training and WT training will be performed twice a week for 12 weeks. The periodization of the training is shown in Chart 1. In the first 3 weeks, each session will consist of 15 bouts of two minutes of exercise with two minutes of passive recovery. After that, the exercise time will progressively increase by one minute every 3 weeks and the recovery period will be decreased, completing, at the end, a maximum volume of 10 bouts of 5 minutes of exercise with 1 minute of passive recovery. The intensity of both exercise groups will be determined by the intensity equivalent to the range of 13 – 15 of Borg scale (Borg scale - 6 to 20) [3]. In addition, the intensity assessment will be done during (each exercise bout) and at the end of each training session. All sessions will be supervised by a qualified kinesiologist with experience in vascular disease rehabilitation.

| **Weeks** | **Bouts** | **Active exercise** | **Total active time** | **Interval** | **Total interval time** | **Total session time** |
| --- | --- | --- | --- | --- | --- | --- |
| 1-3 Week | 15 | 2’ | 30’ | 2’ | 30’ | 60’ |
| 4-6 Week | 14 | 3’ | 42’ | 1’30” | 19’30” | 62’ |
| 7-9 Week | 12 | 4’ | 48’ | 1’ | 11’ | 59’ |
| 10-12 Week | 10 | 5’ | 50’ | 1’ | 9’ | 59’ |

**Chart 1.** Periodization during the 12 weeks of training.

Patients randomized to CG will also attend to meetings with the researcher team twice week during the 12 weeks in order to minimize the effects of the patient's bi-weekly commitment and displacement to the training site, to minimize the influence of the patient-researcher contact and also minimize the convivial effect among the patients themselves, which will occur in the other two groups. During the meetings, patients will perform manual tasks with use of artistic materials, cultural programs, cooking classes and home care, without any exercise component.

All patients included in the study will also receive recommendations to increase levels of physical activity, which is the standard recommendation for clinical treatment for these patients [4].

**Measurements**

For all measurements, patients will be instructed to eat a light meal 2 h beforehand, to avoid caffeinated beverages on the experimental days, and do not perform exercise in the previous 48 hours.

**Primary outcome**

**Ambulatory blood pressure**

Ambulatory BP will be assessed by an oscillometric device (Dyna-MAPA, Cardios, Brasil) programmed to take measurements every 15 min for 24 hours. The recordings will only be accepted if at least 80% of the readings were successfully performed. Patients will be instructed to complete a diary to record the time of sleep, waking and daytime activities. Ambulatory data will be analysed using the averages of 24 h, awake and asleep periods [5].

**Secondary outcomes**

**Cardiovascular Function**

**Office blood pressure**

Systolic and diastolic office BP will be measured by an automatic monitor (HEM-742, Omron Healthcare, Japan). Patients will be resting for ten minutes in a supine position. Three consecutive measurements will be performed with one-minute interval between them, in both arms, and with the appropriate size cuff. The value used will be the average of the last two measures.

**Central blood pressure**

Central BP will be obtained by the pulse wave analysis recorded in the left radial artery using applanation tonometry (SphygmoCor AtcorMedical, Sydney, Australia) and a validated transfer function algorithm provided by the Sphygmocor® software will estimate the central values of systolic, diastolic, and mean BP [6].

**Heart rate variability**

Heart rate variability will be assessed from the beat to beat intervals obtained by a heart rate monitor (V800, Polar Electro, Finland) during 10 min with patient in supine position. Continuous stationary data recorded for at least 5 min will be used for analysis. All analyses will be performed using a software (Kubios HRV, Biosignal Analysis and Medical Imaging Group, Finland) according to the recommendations of the Task Force for heart rate variability [7]. The time-domain parameters (SDNN – standard deviation of all RR intervals, RMSSD – root mean square of the squared differences between adjacent normal RR intervals, pNN50 – the percentage of adjacent intervals over 50ms) and frequency-domain (low frequency, high frequency, and low frequency/high frequency) parameters will be analyzed as previously described [7].

**Arterial stiffness**

The arterial stiffness parameters, such as pulse pressure (difference between systolic and diastolic blood pressure) and augmentation index (the proportion of pulse pressure that is attributed to the reflected pulse wave) will be obtained through applanation tonometry (SphygmoCor, AtCor Medical, Australia), in radial artery. Carotid-femoral pulse wave velocity will be measured by applanation tonometry (Sphygmocor, AtCor Medical, Australia) following the guidelines of the Clinical Application of Arterial Stiffness, Task Force III [6]. Distance between carotid artery to the suprasternal notch and femoral artery to the suprasternal notch will be measured using a standard tape. Electrocardiogram will be simultaneous assessed to obtain heart rate and, according to a “foot-to-foot” method, the time difference between the points will be measured. Then, the distance between the two arteries will be divided by the time difference.

**Vascular function**

Vascular function will be estimated by resting blood flow and flow-mediated dilation measurements obtained by ultrasound technique according to recent recommendations [8]. Images of the brachial arteries will be recorded by a two-dimensional ultrasound with spectral Doppler and linear transducer (Ultra-0122, Philips, The Netherlands). For that, each patient will stay in supine position for at least 20 minutes. After location of the arteries, the transducer will be placed and to attest to the good quality of the arterial pulse obtained, the Doppler sound will be activated.

The contrast resolution, depth, and gain will be adjusted to optimize the longitudinal images of the lumen/arterial wall interface. Insonation angle-corrected at 60°, blood velocity spectra will be simultaneously recorded via the pulsed-wave mode at linear frequencies of 13 and 6.0 MHz, respectively.

Baseline diameter and blood velocity waveforms will be continuously recorded over 120 s. After that, a cuff, placed distal to the image capture, will be inflated with a pressure above 50 mm Hg of the systolic BP measured before the examination. The occlusion will be maintained for five minutes, and, after this period, rapidly released. The image and Doppler captures recordings will be resumed 30 s before deflation and will maintain for 180s after.

The diameters and post-occlusion blood flow velocities will be measured after the release. The vasodilatory capacity will be calculated by the flow-mediated dilation, the percentage of increase in diameter of the brachial artery post occlusion compared to their baseline values.

**Functional Capacity**

**Six-minute walk test**

The six-minute walking test will be performed along a 30-meter-long corridor, as previously described [9,10]. Briefly, patients will be encouraged to “walk at their usual pace for six-minutes and cover as much ground as possible” and rest if necessary. The outcomes will be the onset claudication distance (distance walked when the patients related the occurrence of symptom of intermittent claudication) and six-minute total walking distance (maximum distance achieved by the patient at the end of the test).

**Two-minute step test**

The 2 two-minutes step test will be performed as previously described [11]. Patients will be required to march in place for 2 minutes, being counted the maximum number of steps. The march will be consisted of alternating elevation of the knees to the mean height of the thigh (midpoint between the patella and the anterior superior iliac spine). Patients will be instructed to complete as many steps as possible during the test.

**Walking impairment questionnaire**

The walking impairment questionnaire [12] contains three domains measuring three factors of walking impairment: walking distance, walking speed, and the ability to climb stairs. Patients will be asked how difficult it was to walk in these situations and should answer as “none, slight, some, much or unable”. Each domain is scored on a 0 to 100 scale, where 0 represents extreme limitation and 100 represents no walking long distances difficulties, walking rapidly, or climbing 3 stair flights, respectively.

**Walking estimated limitation calculated by history**

The walking estimated-limitation calculated by history is a four-question questionnaire which the first 3 are related to how long patients can perform the task easily on levels ground and without stopping in different walking speed and the last one is related to speed comparison with their relatives, friends or people at same age. The score (ranges from 0 to 100) is calculated as the sum of the values for the first three, minus one, multiplied by the coefficient for the final (walking speed) questionnaire item [13].

**Handgrip test**

The handgrip test will be performed to evaluate the handgrip strength. The test will be performed using a dynamometer with digital display (EH101, Camry, USA), following the previously protocol [14] that will be calibrated with a scale from 0 to 100 kgf. Patients will be evaluated seated with slightly adducted shoulder, elbow flexed at 90°, forearm and wrist in neutral position. The test will be performed in three attempts in each of the dominant and non-dominant hands, alternately, and the highest value will be used for analysis.

**Baltimore Activity Scale for Intermittent Claudication**

The Baltimore Activity Scale for Intermittent Claudication will be obtained following the previously protocol [15]. It consists of five questions related to the symptoms of intermittent claudication. For each question, patient selects the answer that best describes their symptoms and the level of physical activity. Values range from 0 to 2 points, and the total score is the sum of the points from the 5 questions. The score ranges from 0 to 10, with zero being the lowest levels of physical activity and ten being the highest [15].

**Short Physical Performance Battery**

Functional capacity will also be obtained by the short physical performance battery as previously described [16]. The short physical performance battery is a group of measures that combines the results of gait speed, chair stand, and balance tests. The total score will be calculated from the performance in the three tests, ranging from 0 to 12, with 0 as worst function and 12 as best function.

**Cognition**

Standardized cognitive tasks to quantify executive function and memory will be evaluated as previously described [17]. These assessments will be carried out on paper and include: a) executive function - Test A and B, coding of digit symbols; b) memory: Hopkins verbal learning test (immediate and delayed recovery), forward and backward digit range; c) verbal fluency: task of generating words ("S" and animals).

**Quality of life**

**Vascular quality of life questionnaire - 6**

The quality of life will be evaluated by the vascular quality of life questionnaire VascuQoL-6, as previously described [18]. The questionnaire is composed by six items evaluating the impact of disease on social aspects and capacity to perform daily activities. Each item is scored 1-4. The total score is achieved by summarizing the score on each item, resulting in a score between 6 and 24. Higher value indicates better health status.

**World Health Organization Quality of Life (WHOQOL) brief**

The WHOQOL-brief instrument encircles different aspects of physical and mental health, which encircle the follow dimensions: general health status, functional capacity, physical aspects, pain, vitality, mental health, emotional aspects and social aspects of life. Each answer receives a score, which is added to a constant to determine the different components of quality of life [19].

**Intervening variables**

In order to minimize the possible influences of intervening variables, and to ensure that the changes generated on outcomes will be caused by ACT or WT interventions, medications, dietary pattern, and physical activity levels will be monitored.

To control of medication, each week the researcher will be responsible for filling a container with the medications prescribed for the patient, according to the frequency, time and recommended dosage. At the end of the week (in the second weekly session), the researcher will check if the patient correctly took the medications. Patients will be followed during the 12 weeks period of intervention.

For food monitoring, before and after the intervention period, patient will be asked to fill a food diary for four days of the week, including at least one day of the weekend. Based on this information, it will be possible to analyze changes in the dietary pattern of the patients, as well as to estimate the caloric intake ingested before and after the intervention period.

Finally, patients will use a smartwatch (A300, Polar, Finland) that will estimate their physical activity levels. The POLAR A300 is a monitor coupled with a 3D accelerometer that records patient’s movements. Through it, it is possible to analyze the frequency, intensity and regularity of the movements and, consequently, to determine the physical activity levels. Patients will use this monitor for one week before the first evaluation and after the last week of training, allowing to identify possible changes in physical activity level with ACT, WT and CG interventions.

**Statistical analysis**

**Power and sample size**

The sample size was determined by the a priori specific sample calculation for clinical trials involving parallel groups, in this case two-way ANOVA with repeated measures. Using GPower 3.1.9.2 software it was considered an effect size of 0.25; α of 0.05; power (1 - β) of 0.80; correlation coefficient between repeated measures of 0.6; non-spherical correction (ɛ) of 1 and three groups two measures. Thus, the minimum total size reached 36 subjects (12 per group) with 83% power. Considering the possibility of sample loss, the sample was inflated by 20%, resulting in a sample of 45 patients (15 per group).

**References**

1. Aboyans V, Ricco JB, Bartelink MEL, Bjorck M, Brodmann M, Cohnert T, et al. 2017 ESC Guidelines on the Diagnosis and Treatment of Peripheral Arterial Diseases, in collaboration with the European Society for Vascular Surgery (ESVS): Document covering atherosclerotic disease of extracranial carotid and vertebral, mesenteric, renal, upper and lower extremity arteriesEndorsed by: the European Stroke Organization (ESO) The Task Force for the Diagnosis and Treatment of Peripheral Arterial Diseases of the European Society of Cardiology (ESC) and of the European Society for Vascular Surgery (ESVS). Eur Heart J. 2018; 39(9): 763-816. <https://doi.org/10.1093/eurheartj/ehx095> PMID:

2. Gardner AW, Skinner JS, Cantwell BW, Smith LK. Progressive vs single-stage treadmill tests for evaluation of claudication. Med Sci Sports Exerc. 1991; 23(4): 402-428.

3. Borg GAV. Psychophysical bases of perceived exertion. Med Sci Sports Exerc. 1982; 14(5): 377-381.

4. Brook RD, Appel LJ, Rubenfire M, Ogedegbe G, Bisognano JD, Elliott WJ, et al. Beyond medications and diet: alternative approaches to lowering blood pressure: a scientific statement from the american heart association. Hypertension. 2013; 61(6): 1360-83.

5. Parati G, Stergiou G, O’Brien E, Asmar R, Beilin L, Bilo G, et al. European Society of Hypertension practice guidelines for ambulatory blood pressure monitoring. J Hypertens. 2014; 32(7): 1359-1366.

6. Van Bortel LM, Duprez D, Starmans-Kool MJ, Safar ME, Giannattasio C, Cockcroft J, et al. Task Force III: recommendations for user procedures. Am J Hypertens. 2002; 15(5): 445-452.

7. Task Force of the European Society of Cardiology, The North American Society of Pacing and Electrophysiology. Heart rate variability: standards of measurement, physiological interpretation and clinical use. Task Force of the European Society of Cardiology and the North American Society of Pacing and Electrophysiology. Circulation. 1996; 93(5): 1043-1065.

8. Thijssen DH, Black MA, Pyke KE, Padilla J, Atkinson G, Harris RA, et al. Assessment of flow-mediated dilation in humans: a methodological and physiological guideline. Am J Physiol Heart Circ Physiol. 2011; 300(1): H2-12.

9. Montgomery PS, Gardner AW. The clinical utility of a six-minute walk test in peripheral arterial occlusive disease patients. J Am Geriatr Soc. 1998; 46(6): 706-711.

10. Ritti-Dias RM, Sant'anna FDS, Braghieri HA, Wolosker N, Puech-Leao P, Lanza FC, et al. Expanding the use of six-minute walking test in patients with intermittent claudication. Ann Vasc Surg. 2021; 70: 258-262.

11. Węgrzynowska-Teodorczyk K, Mozdzanowska D, Josiak K, Siennicka A, Nowakowska K, Banasiak W, et al. Could the two-minute step test be an alternative to the six-minute walk test for patients with systolic heart failure? Eur J Prev Cardiol. 2016; 23(12): 1307-1313.

12. Ritti-Dias RM, Gobbo LA, Cucato GG, Wolosker N, Jacob Filho W, Santarém JM, et al. Translation and validation of the walking impairment questionnaire in Brazilian subjects with intermittent claudication. Arq Bras Cardiol. 2009; 92(2): 136-149.

13. Cucato GG, Correia MdA, Farah BQ, Saes GF, Lima AHdA, Ritti-Dias RM, et al. Validation of a brazilian portuguese version of the walking estimated-limitation calculated by history (WELCH). Arq Bras Cardiol. 2016; 106(1): 49-55.

14. Silva GO, Farah BQ, Germano-Soares AH, Andrade-Lima A, Santana FS, Rodrigues SL, et al. Acute blood pressure responses after different isometric handgrip protocols in hypertensive patients. Clinics. 2018; 73: e373.

15. Gardner AW, Montgomery PS. The baltimore activity scale for intermittent claudication: a validation study. Vasc Endovascular Surg. 2006; 40(5): 383-391.

16. Guralnik JM, Simonsick EM, Ferrucci L, Glynn RJ, Berkman LF, Blazer DG, et al. A short physical performance battery assessing lower extremity function: association with self-reported disability and prediction of mortality and nursing home admission. J Gerontol. 1994; 49(2): M85-94.

17. Carson N, Leach L, Murphy KJ. A re-examination of Montreal Cognitive Assessment (MoCA) cutoff scores. Int J Geriatr Psychiatry. 2018; 33(2): 379-388.

18. de Almeida Correia M, Andrade-Lima A, Mesquita de Oliveira PL, Domiciano RM, Ribeiro Domingues WJ, Wolosker N, et al. Translation and validation of the brazilian-portuguese short version of vascular quality of life questionnaire in peripheral artery disease patients with intermittent claudication symptoms. Ann Vasc Surg. 2018; 51(1): 48-54.

19. The World Health Organization quality of life assessment (WHOQOL): Position paper from the World Health Organization. Soc Sci Med. 1995; 41(10): 1403-1409.
